# Characterization of Histone Genes from the Bivalve Lucina Pectinata

**DOI:** 10.3390/ijerph15102170

**Published:** 2018-10-02

**Authors:** Ingrid M. Montes-Rodríguez, Yesenia Rodríguez-Pou, Ricardo R. González-Méndez, Juan Lopez-Garriga, Alexander J. Ropelewski, Carmen L. Cadilla

**Affiliations:** 1 Comprehensive Cancer Center University of Puerto Rico. P.O. BOX 363027, San Juan 00936-3027, Puerto Rico; imontes@cccupr.org; 2 Department of Biochemistry, School of Medicine, University of Puerto Rico-Medical Sciences Campus, P.O. Box 365067, San Juan 00936-5067, Puerto Rico; yesenia.rodriguez8@upr.edu; 3 Department of Radiological Sciences, School of Medicine, University of Puerto Rico-Medical Sciences Campus, P.O. Box 365067, San Juan 00936-5067, Puerto Rico; ricardo.gonzalez7@upr.edu; 4 Department of Chemistry, Faculty of Arts and Sciences, University of Puerto Rico—Mayagüez Campus, P.O. Box 9019, Mayagüez 00681-9019, Puerto Rico; juan.lopez16@upr.edu; 5 Pittsburgh Supercomputing Center 300 South Craig Street Pittsburgh PA 15213; ropelews@psc.edu

**Keywords:** histone gene, gene variants, mollusk, gene evolution, bioinformatics, next-generation sequencing, Sanger sequencing

## Abstract

*Lucina pectinata* is a clam that lives in sulfide-rich environments and houses intracellular sulfide-oxidizing endosymbionts. To identify new *Lucina pectinata* proteins, we produced libraries for genome and transcriptome sequencing and assembled them de novo. We searched for histone-like sequences using the *Lucina pectinata* histone H3 partial nucleotide sequence against our previously described genome assembly to obtain the complete coding region and identify H3 coding sequences from mollusk sequences in Genbank. *Solen marginatus* histone nucleotide sequences were used as query sequences using the genome and transcriptome assemblies to identify the *Lucina pectinata* H1, H2A, H2B and H4 genes and mRNAs and obtained the complete coding regions of the five histone genes by RT-PCR combined with automated Sanger DNA sequencing. The amino acid sequence conservation between the *Lucina pectinata* and *Solen marginatus* histones was: 77%, 93%, 83%, 96% and 97% for H1, H2A, H2B, H3 and H4, respectively. As expected, the H3 and H4 proteins were the most conserved and the H1 proteins were most similar to H1′s from aquatic organisms like *Crassostrea gigas, Aplysia californica, Mytilus trossulus* and *Biomphalaria glabrata*. The *Lucina pectinata* draft genome and transcriptome assemblies, obtained by semiconductor sequencing, were adequate for identification of conserved proteins as evidenced by our results for the histone genes.

## 1. Introduction

In eukaryotes and some archaebacteria, DNA is found as a nucleoprotein complex called chromatin, associated with histones that allows for the high levels of compaction of genomic DNA within the limited space of the cell nucleus [1]. Histones are basic proteins, four of them (H3, H4, H2A and H2B) form the histone octamer that DNA winds around to form the nucleosome, the fundamental structural unit of chromatin [2]. A fifth lysine-rich histone (H1 and related proteins) binds the nucleosome at the entry and exit sites of the DNA, locking the DNA into place and allowing the development of higher order structures. Histones also play a major role in modulating changes in chromatin structure, impacting gene regulation, mostly through their post-translational modifications [3].

The core histones exist as dimers; they all possess the histone fold domain: three alpha helices linked by two loops. This helical structure allows for interaction between distinct dimers. The histone H1 family stands out among histone for being the most diverse [3]. Though they are relatively conserved, especially in their central globular domain, which consists of a 3-helix “winged helix” fold containing a classical helix-turn-helix motif, they are the most variable histones across different species [4]. Their sequence complexity may be related to subtype functional differentiation [4]. Much has been learned about histone H1 from studies on purified chromatin fibers. Ionic extraction of these linker histones from native chromatin promotes its unfolding under hypotonic conditions [5]. It is still unknown if H1 promotes a solenoid-like chromatin fiber, in which the exposed linker DNA is condensed, or if it merely promotes a change in the angle of adjacent nucleosomes without affecting the linker’s length [6]. Although the long-term evolution of the H1 family has been studied in deuterostomes in previous years, the invertebrate H1 proteins, especially in animals such as mollusks, still remain less studied [7]. It has been proposed that H1 histone genes are subject to birth-and-death evolution with strong purifying selection, which may explain the existence of replication-dependent (RD) and replication-independent H1 isoforms [7].

The objective of this work is to characterize the histone complement and better understand the evolution of proteins like the H1 family using the clam *Lucina pectinata* (*L. pectinata*) as a model system for invertebrates. *L. pectinata* is a bivalve that lives in sulfide-rich environments and houses intracellular sulfide-oxidizing endosymbiotic bacteria. Since it has been proposed that histone H1 isoforms may arise by functional differentiation, the ability of this clam to exist in its tropical but extreme environment may have given rise to different subtypes.

In order to identify new proteins in this organism, a draft genome was assembled from a genomic library and characterized using semiconductor sequencing chemistry [8]. To validate the genome assembly and identify histone genes for *L. pectinata*, a search for Histone-like sequences was performed using the known partial coding sequence of histone H3 from *L. pectinata* and the nucleotide sequences of the Histone H1, H2A, H2B, H3 and H4 from the clam *Solen marginatus* (*S. marginatus*) as query sequences using the draft genome as library [1].

For evaluation of the evolutionary aspects of this study, we focused on the *L. pectinata* histone H1 variant sequences (H1_A and H1_B) that were obtained, to determine if these variants have a common ancestor found in other marine organisms and to study their evolution among different species. These findings contribute to the study of functional differentiation of the histone H1 family among mollusk species. 

## 2. Materials and Methods

### 2.1. Biological

Ethics statement: The clam *Lucina pectinata* houses symbiotic bacteria and lives in shallow sea waters of mangrove swamps in Southwestern Puerto Rico, whose sediments contain high sulfide concentrations. Juvenile *L. pectinata* clams were purchased from a local fisherman in the town of Cabo Rojo, PR. Typically, when our collaborating fisherman harvests the clams in the mangroves of Cabo Rojo for research purposes, they are kept in buckets filled with mangrove mud and seawater until they were picked up the same day they were harvested. *L. pectinata* is not an endangered or protected species, hence, no specific permits were required to obtain the clams since they are a local food item. Clams were transported in seawater containing mud extracted from the site where they were harvested, until either used for isolation and purification of DNA or RNA or until the tissues were harvested and snap frozen for subsequent protein isolation.

### 2.2. DNA and RNA Isolation

Genomic DNA (gDNA) from *L. pectinata* was extracted from 100 mg of mantle tissue, in order to avoid endosymbiont contamination, using the QIAGEN Genomic-tip 100/G (QIAGEN, Inc. Germantown, MD, USA) following the manufacturer’s instructions for DNA isolation from animal tissue. Total gDNA was quantified with the Nanodrop 2000c (Thermo Fisher Scientific, Inc. Wilmington, DE, USA), and the concentration of double stranded DNA with the Qubit dsDNA HS Assay Kit from Invitrogen in the Qubit® 2.0 Fluorometer (Invitrogen, Carlsbad, CA, USA), as recommended by the manufacturer’s instructions.

Several clams were carefully dissected within 24 h after clam harvest for RNA isolation. The rest of the clams were put in a fish-tank with seawater and a pump filter. They were fed phytoplankton twice a week. At 108 days they were snap frozen in liquid nitrogen and stored at −80 °C until processed. Ctenidia RNAs were isolated from three clams harvested from their natural environment and three clams kept in a fish tank for 108 days. RNA was extracted from the ctenidia tissue using TRIzol reagent (Sigma-Aldrich, St. Louis, MO, USA), following the modified procedure of RNA isolation described by Chomczynski [9], as recommended by the manufacturer. To improve the A_260_/A_230_ ratio of the RNA samples, RNAs were extracted with 1-Butanol followed with two consecutive diethyl ether extractions [10], in order to remove traces of TRIzol reagent.

### 2.3. Whole Genome Sequencing

A genomic library for Ion Proton sequencing was created using the IonPlus Fragment Library kit from Life Technologies (Carlsbad, CA) , according to the Ion Xpress™ Plus gDNA Fragment Library Preparation protocol following the manufacturer’s instructions (Publication Number MAN0009847). Briefly, 1µg of high molecular weight mantle gDNA was fragmented by sonication using the Bioruptor® UCD-200 sonication device (Denville, NJ, USA). After fragmentation, the DNA was end-repaired and purified followed by the adaptor ligation, nick-repair, and purification of the ligated DNA as indicated in the manufacturer’s manual. The ligated DNA was size-selected using the Pippin Prep^TM^ instrument (SAGE Science, Beverly, MA, USA) with the pre-cast 2% agarose gel (ethidium bromide-stained) cassettes and marker B (Sage Science, Beverly, MA). Then, the sample was purified with 1.5× volumes of Agencourt AMPure® XP Reagent (Beckman Coulter Inc., Indianapolis, IN) and its concentration determined by quantitative PCR (qPCR) using the Ion Library Quantitation Kit (Life Technologies, Carlsbad, CA, USA), as recommended by the manufacturer. The sample was amplified with the Platinum® PCR Super Mix High Fidelity (Invitrogen, Carlsbad, CA) reaction mixture and the Library Amplification Primer Mix, supplied by the IonPlus Fragment Library kit and purified. Afterwards, the library was analyzed on the Agilent® 2100 Bioanalyzer® instrument (Agilent Technologies, Santa Clara, CA) with an Agilent High Sensitivity DNA Kit to evaluate size distribution of the final library and the library concentration was determined by qPCR with the Ion Library Quantitation Kit (Life Technologies, Carlsbad, CA, USA). The DNA fragment size distribution was ca. 200 base pairs (bp). The emulsion PCR step and enrichment of the library were carried out in the Ion OneTouch™ 2 System and in the Ion OneTouch™ ES System (Life Technologies, Carlsbad, CA), respectively, using the Ion PI™ Template OT2 200 Kit v3 from Life Technologies following the Ion PI™ Template OT2 200 Kit v3 User Guide (Publication # MAN0009133). Next, the sample was run in the Ion Proton™ Sequencer using the Ion PI^TM^ Chip V2 (Life Technologies, Carlsbad, CA). The sample was prepared using the Ion PI^TM^ Sequencing 200 Kit v3 (Life Technologies, Carlsbad, CA) following the Ion PI^TM^ Sequencing 200 Kit v3 User Manual (Publication Number MAN0009136) and run in the Ion Proton^TM^ Sequencer (Life Technologies, Carlsbad, CA).

The sequence read data was analyzed on the Pittsburgh Supercomputing Center’s Blacklight system. The sequence reads were submitted to the NCBI Sequence Read Archives (SRA) and assigned Run number SRR7822584, SRA Sample SRS3768289 and SRA Study SRP161589. To create a draft de novo assembly, an unpaired low-coverage Ion Torrent dataset was selected according to the output and quality of data available. The reads were quality control trimmed and filtered using the Sickle Windowed Adaptive Trimming Tool, discarding reads with <75 bases in length and used a quality score threshold of 15. Then, the trimmed data was assembled with MIRA Version 4.0.2 as described [8]. The genome assembly was submitted to the NCBI Genbank database, with submission number SUB4511435, the GenBank accession number is pending.

### 2.4. RNA Sequencing

Transcriptome libraries were made using the Ion Total RNA-Seq Kit v2 from Life Technologies following the manufacturer’s instructions (Publication Number 4476286). The quality of RNA was verified by running the Agilent RNA 6000 Nano Kit in the Bioanalyzer® 2100 instrument following the RNA 6000 Nano Kit user manual. Briefly, 0.5–1 µg of total RNA were used as starting material. We reduced ribosomal RNA (rRNA) content using the Low Input RiboMinus^TM^ Eukaryote System V2 (Life Technologies, Carlsbad, CA), following the kit user guide (Publication Number MAN 0007160). The qualitative analysis of rRNA removal was assessed comparing the total RNA profiles before and after the removal of rRNA using the Agilent RNA 6000 Pico Kit in the Bioanalyzer® 2100 instrument (Agilent Technologies, Santa Clara, CA, USA) as recommended. In the clam *Lucina pectinata*, the 28SrRNA fragment is not observed due to presence of a break point in the rRNA structure which converts the 28S rRNA to two fragments that migrate similarly to the 18S rRNA in gel electrophoresis. Hence, we assessed the reduction of rRNA content by analysis of the 18S rRNA peaks content versus the lower molecular weight abundant RNAs. Bioanalyzer traces showed that five out of the six RNA samples used for RNA sequencing had rRNA peaks eliminated and one of the samples had a 6.3× reduction of rRNA content, demonstrating the utility of the RiboMinus kit employed for rRNA reduction in this type of organism. Then, these samples were fragmented by RNase III digestion for 3 min at 37 °C and purified following the Ion Total RNA-Seq Kit v2 (Life Technologies, Carlsbad, CA) manual specifications. Sample yield and size distribution were assessed using the RNA 6000 Pico Kit with the Agilent 2100 Bioanalyzer® instrument. Two RNA samples from each environment (4 in total) were barcoded using Ion Xpress^TM^ RNA-Seq Barcode 1–16 Kit (Life Technologies, Carlsbad, CA). The remaining two RNA samples (one from each environment) were run on individual IonProton PI chips. Emulsion PCR and enrichment steps were carried out in the Ion OneTouch™ 2 System and the Ion OneTouch™ ES System (Life Technologies, Carlsbad, CA), respectively, using the Ion PI™ Template OT2 200 Kit v3 from Life Technologies following the Ion PI™ Template OT2 200 Kit v3 User Guide (Publication Number MAN0009133). Each sample was run in the Ion Proton™ Semiconductor Sequencer using the Ion PI^TM^ Chip V2 (Life Technologies, Carlsbad, CA). The samples were prepared using the Ion PI^TM^ Sequencing 200 Kit v3 (Life Technologies, Carlsbad, CA) following the user manual (Publication Number MAN0009136). A total of three chips (one chip with the four barcorded libraries and the other two individual samples) were loaded and run in the Ion Proton^TM^ Sequencer (Life Technologies, Carlsbad, CA). Sequence reads from all chips were combined in order to assemble a reference transcriptome de novo.

The Ion Torrent data was analyzed on the Pittsburgh Supercomputer Center’s Blacklight system, a high-performance computing system available through the National Science Foundation (NSF) Extreme Science and Engineering Discovery Environment (XSEDE) Program. A transcriptome reference was assembled de novo using Trinity software version r2014-04-13p1 [11]. All six single ended read samples were combined to create a single data file for the assembly run. The default parameters for single ended read sequences were used to create the assembly [11]. Raw read sequence data were deposited in NCBI’s sequence read archive (SRA) database: BioProject PRJNA282817. The Transcriptome Shotgun Assembly project has been deposited at DDBJ/EMBL/GenBank under the accession GGWH00000000. The version described in this paper is the first version, GGWH01000000.

### 2.5. Sequence Similarity Searches and Validation of Semiconductor Sequencing Results

In order to find the histone sequences in our draft genome, we searched for homologous sequences from an organism that was evolutionary close to *L. pectinata*. For this reason, we used the histone nucleotide sequences from another clam, *S. marginatus* using the Basic Local Alignment and Search Tool (BLAST) for nucleotide searches, BLASTN [12,13,14], optimized for somewhat similar sequences, using the known gene nucleotide sequences of the histone 1 (H1) (GenBank accession number FJ595834.1), histone 2A (H2A) (GenBank accession number FJ595835.1), histone 2B (H2B) (GenBank accession number FJ595836.1), histone 3 (H3) (GenBank accession number FJ595837.1) and histone 4 (H4) (GenBank accession number FJ595838.1) from the clam *Solen marginatus* [1] and the partial coding sequence of the *L. pectinata* histone H3 (GenBank accession number GQ980264.1) as query sequences. We chose the sequences that produced a good match (with identities >68%) and searched for open reading frames (ORF) using the ORF Finder tool from NCBI performing a six-frame translation of each of the nucleotide sequences [15]. Each of the translated sequences of adequate length for each type of histone were analyzed with the protein BLAST (BLASTP) tool, using default parameters against the non-redundant protein database [14,15]. We selected those sequences that produced a complete protein (comparing histone sizes across different organisms) and had histone conserved domains. In order to amplify these regions, we designed forward and reverse primers for each of these nucleotide sequences using the Primer3Plus software [16] ensuring that the complete coding region was included in the regions amplified with both primers (see Table 1). The RT-PCRs were carried out using the One®Step RT-PCR kit (QIAGEN, Germantown, MD) following the manufacturer’s instructions. The reaction consisted of 1X OneStep Buffer, 0.4 mM dNTP mix, 0.6 mM of each primer (forward and reverse), 5 µL of Q-solution, 1 µL QIAGEN Enzyme Mix in a total volume of 25 µL and 135 ng of *L. pectinata* ctenidia RNA. The cycling parameters were: 50 °C for 30 min, 95 °C for 15 min, 40 cycles of 94 °C for 30 s, 60 °C for 45 s and 72 °C for 1 min, followed by 72 °C for 10 min. The RT-PCR products were evaluated in a 1% agarose gel, purified using QIAGEN PCR purification kit and sequenced on both strands in an ABI 310 automated DNA sequencer using dye terminator chemistry (Big Dye V3 DyeTerminator Sequencing kit, Applied Biosystems, Foster City, CA).

### 2.6. Histone Protein Extraction and Electrophoresis

We isolated the histone proteins from ctenidia and mantle tissues using a Histone Extraction kit (Abcam) according to the manufacturer’s instructions. First, the tissue sample was cut into small pieces (1–2 mm^3^) with a scalpel, followed by homogenization in one volume of Pre-Lysis buffer, (ca. 200 mg/mL) in a glass Dounce homogenizer. All centrifugation steps were carried out in a refrigerated microcentrifuge (Eppendorf 5830R, Eppendorf North America, Hauppauge, NY, USA). The homogenate was transferred to a 15 mL conical tube and centrifuged at 3000 rpm for 5 min at 4 °C. The supernatant was removed and discarded and the pellet resuspended in three volumes of Lysis Buffer, incubated on ice for 30 min, followed by centrifugation at 12,000 rpm for 5 min at 4 °C. The supernatant (containing acid-soluble proteins) was transferred into a new vial. Then, 0.3 volumes of the Balance-DTT Buffer were added immediately to the supernatant (e.g., 0.3 mL of Balance-DTT Buffer to 1 mL of supernatant). The samples were quantified in a Nanodrop 2000c by measuring the A_280_.

Histone proteins were analyzed in a discontinuous sodium dodecyl sulfate (SDS) Polyacrylamide Gel Electrophoresis (PAGE) buffer system using the Mini-PROTEAN® System (BIO- RAD, Hercules, CA, USA) as recommended by Thoma and Kornberg [17]. We loaded 20 µg of each sample, including calf thymus histones as controls (Sigma Aldrich, St. Louis, MO), which were dissolved in water at a final concentration of 10 µg/µL. The gel was run for 120 min at 0.3 A, constant current, fixed for 1 h in 50% methanol and 10% glacial acetic acid and stained (0.1% Coomassie Brilliant Blue R-250, 50% methanol and 10% glacial acetic acid) for 20 min with gentle agitation. The gel was destained in a solution containing 40% methanol and 10% glacial acetic acid.

### 2.7. Selection of Sequences from Different Species Related To The Histone H1_A Protein Sequence

The *L. pectinata* H1_A and B amino acid sequences were derived from their nucleotide sequences. To determine the conservation of the H1_A amino acid sequence among species, a BLAST search was performed using the Universal Protein Resource (UniProt) Knowledge Base (UniProtKB) and UniProtKB/SWISS-PROT databases, where curated sequences selected ranged in identity from 80 to 50%. The species selected are shown in Table S1 of the supplementary section. Once the species were selected, these sequences were downloaded as a text file in FASTA format, the *L. pectinata* H1_B query sequence was added, and a multiple sequence alignment was made.

### 2.8. Multiple Sequence Alignment of Histone H1 Proteins from Various Species and Phylogenetic Analysis of Histone H1 Proteins

We employed the Pittsburgh Supercomputing Center’s BioU/Galaxy server to perform phylogenetic analysis. For the multiple sequence alignment of selected H1 proteins, a ClustalW alignment was performed using a BLOSUM30 matrix and a gap open/extension penalty of 10.0 and 0.1, respectively. The alignments were visualized on the server and using Jalview [18]. The multiple sequence alignment was trimmed using the trimAl tool using the heuristic ‘automated1′. Using the trimmed alignment, a consensus phylogenetic tree with 100 bootstrap replicates was generated using the PHYLIP suite of tools [19]. To generate multiple data sets of our input data, we employed the bootstrap and Seqboot using 100 replicates. To estimate phylogenies from protein amino acid sequences by maximum likelihood from our bootstrapped protein dataset, ProML was executed using 100 replicates, jumbling the sequence order (arranging our sequences in random order instead of arranging them in the order they were uploaded). Finally, the Consense tool in PHYLIP was used to generate an unrooted consensus tree using an extended majority rule [19].

The consensus tree was downloaded in Newick format, and visualized using the Interactive Tree of Life (iTOL, [20]) server.

## 3. Results

### 3.1. Sequence Similarity Searches Identify Lucina Pectinata Histone Genes in Genome Assembly

Our strategy to search for histone genes relied on the fact that histones have been highly conserved in evolution. H4 and H3 are highly conserved while H1, H2A and H2B are less conserved [21]. However, at the nucleotide level, the sequence conservation could be lower due to degeneration of the genetic code. We used the histone nucleotide sequences from *S. marginatus* to retrieve at least one nucleotide sequence for each histone gene with relatively high identities even at the nucleotide level: 69%, 77%, 77%, 82% and 80% for H1, H2A, H2B, H3 and H4, respectively (see Table 2).

**Figure 1:**
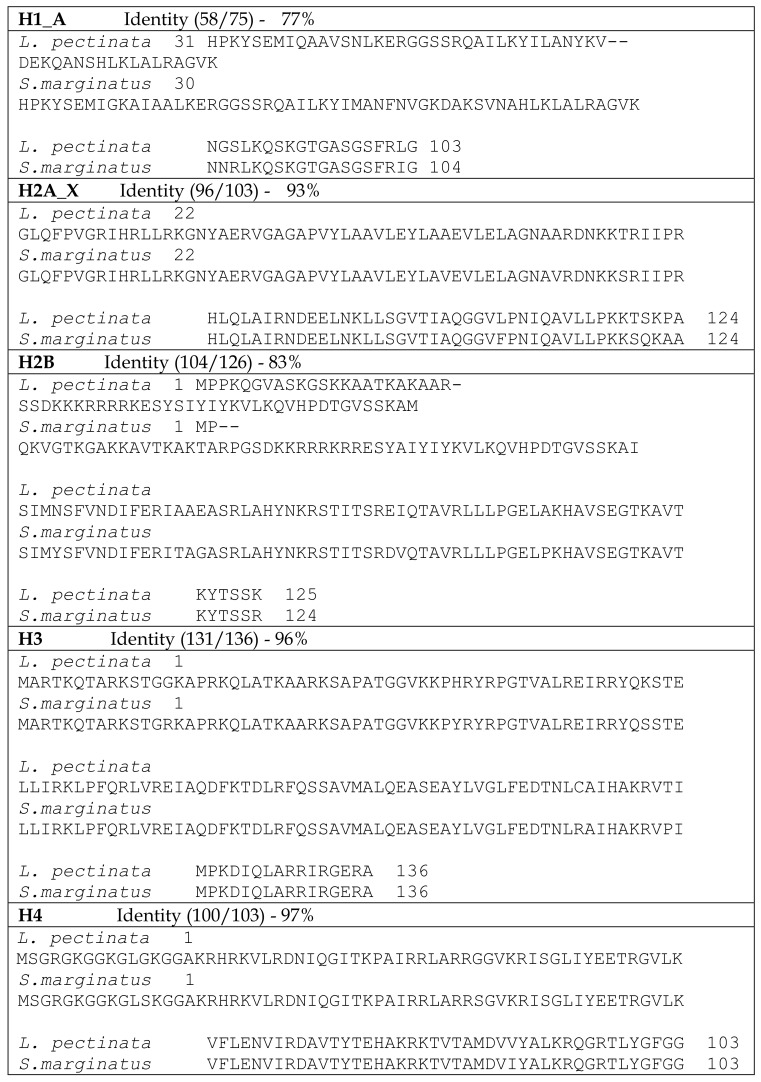
BLASTP alignments between histone protein sequences from *Lucina pectinata* and *Solen marginatus*. Areas of sequence identity are highlighted in gray.

We also compared the predicted histone amino acid sequences from *L. pectinata* with those of *S. marginatus* (see Figure 1) and, as expected, sequence conservation at the protein level was even higher: 77%, 93%, 83%, 96% and 97% for H1_A, H2A, H2B, H3 and H4, respectively. The overall properties of the *L. pectinata* histones were very similar to the *S. marginatus* counterparts (Table 3), having similar amino acid content, pI and molecular weight.

**Figure 2:**
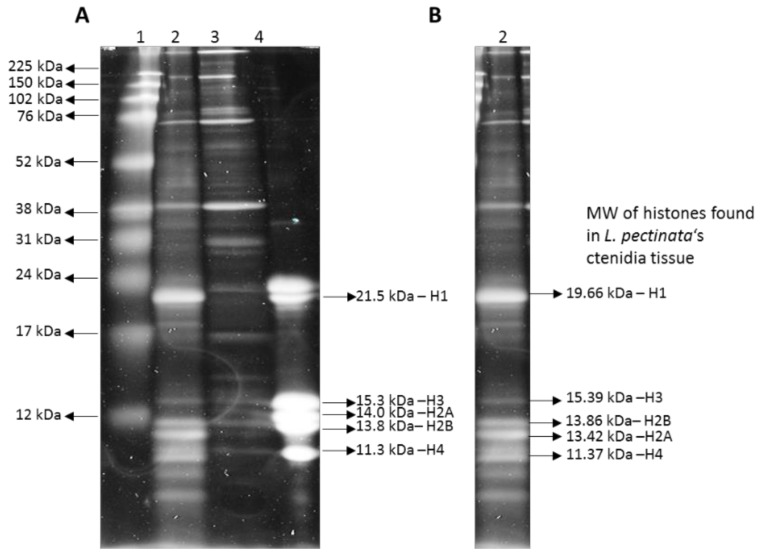
Discontinuous SDS-PAGE gel with isolated histones from *Lucina pectinata*’s ctenidia and mantle tissues. (**A**) Lane 1—Broad Range rainbow protein ladder (Amersham), Lanes 2 and 3—histones from *L. pectinata* tissue; (2) ctenidia; (3) mantle; (4) histones from Calf Thymus (Sigma). (**B**) Lane with histones extracted from *L. pectinata* ctenidia (lane 2 in section A) with the predicted molecular weights of H1, H2A, H2B, H3 and H4.

The discontinuous SDS-PAGE analysis showed that there are additional protein bands for the histones isolated from the mantle tissue (Figure 2). This could be due to the presence of histone variants, since many organisms possess multiple copies of histone gene clusters, which could give rise to non-allelic variation [21,22], or post-translational modifications, degradation or co-purification of abundant non-histone proteins.

**Figure 3:**
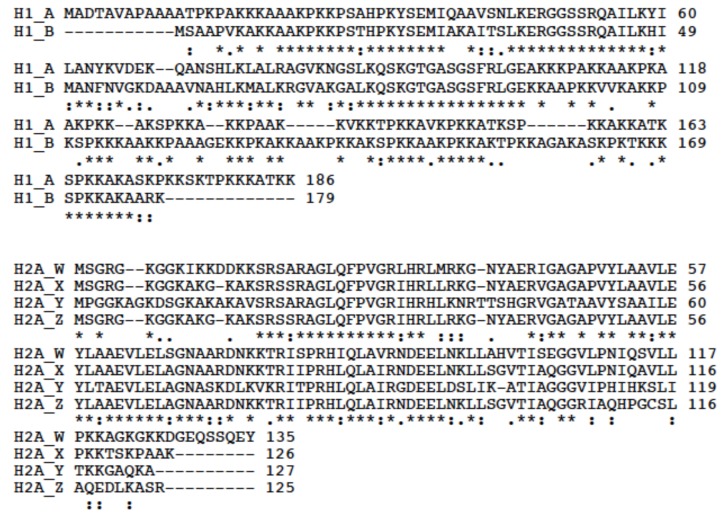
Multiple Alignments of the two *Lucina pectinata* H1 and the four H2A amino acid sequences. These alignments were generated using cluster analysis of the pairwise alignments with the CLUSTAL Omega tool available at https://www.uniprot.org/align/.

Furthermore, sequence analysis verified the presence of two H1 and four H2A variants, most were verified by Sanger dideoxy terminator sequencing of RT-PCR products, resulting in an identical match with those sequences obtained from the draft genome and transcriptome assemblies. Alignments of the two H1 (H1_A and H1_B) and four H2A (H2A_W, H2A_X, H2A_Y and H2A_Z) proteins identified are shown in Figure 3, where the two H1 proteins are only 49.8% identical, with 101 identical and 31 similar amino acids, whereas the four H2A proteins had an overall identity of 42.7%, with 59 identical and 30 similar residues.

**Figure 4:**
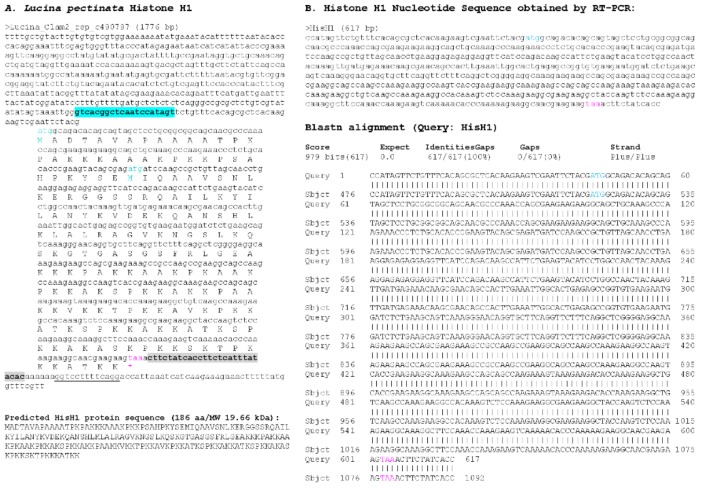
Histone H1_A sequence found in *Lucina pectinata* genome assembly. (**A**) One of the two H1-like nucleotide sequences obtained from the BLASTN searches against the *L. pectinata* genome assembly using the H1 nucleotide sequence from *S. marginatus* is shown, including the coding region, the predicted protein sequence and its molecular weight (MW). (**B**) The nucleotide sequence obtained by Sanger sequencing of RT-PCR products followed by the BLASTN alignment of the cDNA to the genomic sequence, showing a perfect match between the two sequences. Primers used in RT-PCR are highlighted in cyan and grey. Start (cyan) and stop (pink) codons are indicated.

**Figure 5:**
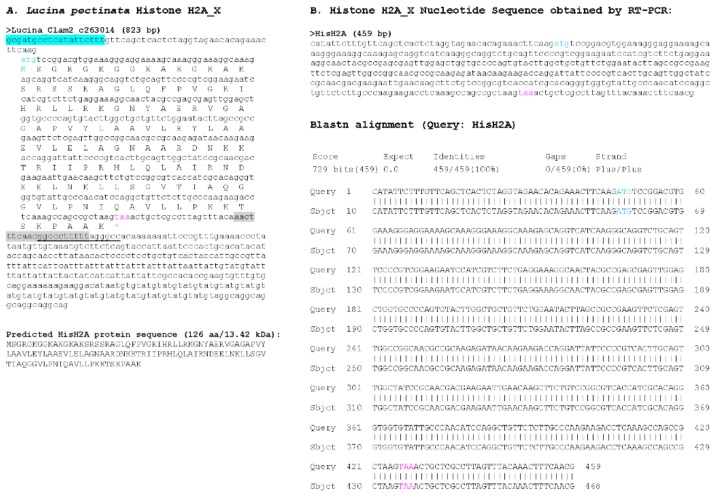
Histone H2A_X sequence found in *Lucina pectinata* genome assembly. (**A**) One of the four H2A-like nucleotide sequence obtained from the BLASTN searches against the *L. pectinata* genome assembly using the H2A nucleotide sequence from *S. marginatus* is shown, including the coding region, the predicted protein sequence and its molecular weight (MW). Primers used in RT-PCR are highlighted in cyan and grey. Start (cyan) and stop (pink) codons are indicated. (**B**) The nucleotide sequence obtained by Sanger sequencing of RT-PCR products followed by the BLASTN alignment of the cDNA to the genomic sequence, showing a perfect match between the two sequences.

**Figure 6:**
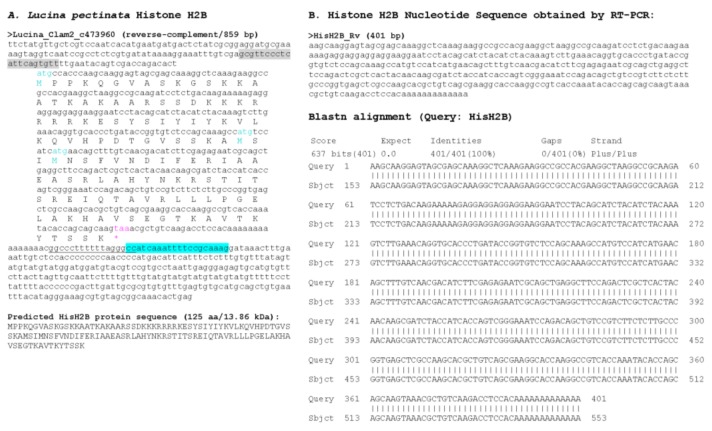
Histone H2B sequence found in *Lucina pectinata* genome assembly. (**A**) Histone H2B nucleotide sequence obtained from the BLASTN searches against the *L. pectinata* genome assembly using the H2B nucleotide sequence from *S. marginatus* is shown, including the coding region, the predicted protein sequence and its molecular weight (MW). (**B**) The nucleotide sequence obtained by Sanger sequencing of RT-PCR products followed by the BLASTN alignment of the cDNA to the genomic sequence, showing a perfect match between the two sequences. Primers used in RT-PCR are highlighted in cyan and grey. Start (cyan) and stop (pink) codons are indicated.

**Figure 7:**
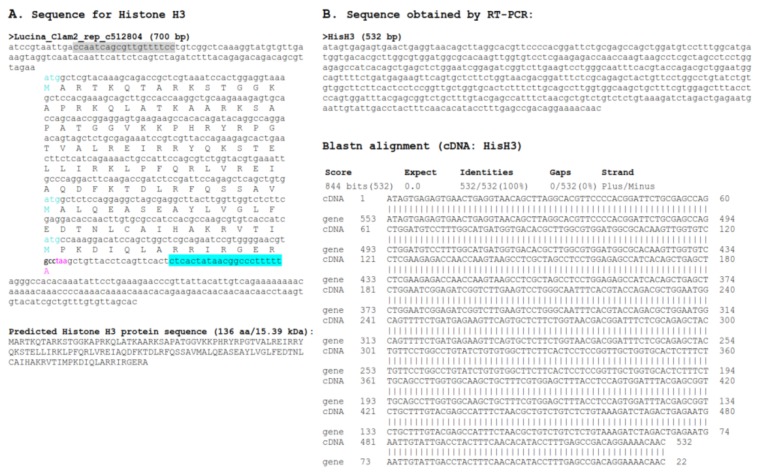
Histone H3 sequence found in *Lucina pectinata* genome assembly. (**A**) The H3 nucleotide sequence obtained from the BLASTN searches against the *L. pectinata* genome assembly using the H3 nucleotide sequences from *S. marginatus* and the partial coding region sequence of H3 from *L. pectinata* is shown with the ORF translation, the predicted protein sequence and its molecular weight (MW). (**B**) The sequence obtained by Sanger sequencing of RT-PCR products by the BLASTN alignment to the genomic sequence, showing a perfect match between the two sequences. Primers used in RT-PCR are highlighted in cyan and grey. Start (cyan) and stop (pink) codons are indicated.

**Figure 8:**
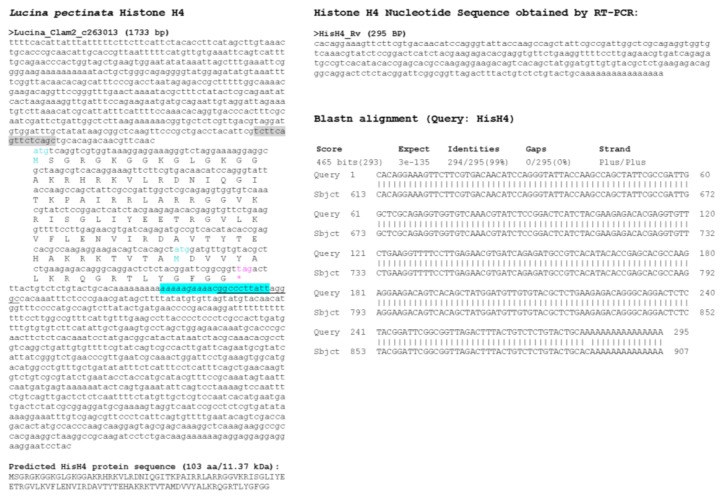
Histone H4 sequence found in *L. pectinata* genome assembly. (**A**) The H4 nucleotide sequence obtained from the BLASTN searches against the *L. pectinata* genome assembly using the H4 nucleotide sequences from *S. marginatus* is shown, with the ORF translation, the predicted protein sequence and its molecular weight (MW). Primers used in RT-PCR are highlighted in cyan and grey. Start (cyan) and stop (pink) codons are indicated. (**B**) The sequence obtained by Sanger sequencing of RT-PCR products by the BLASTN alignment to the genomic sequence, showing a perfect match between the two sequences.

**Figure 9:**
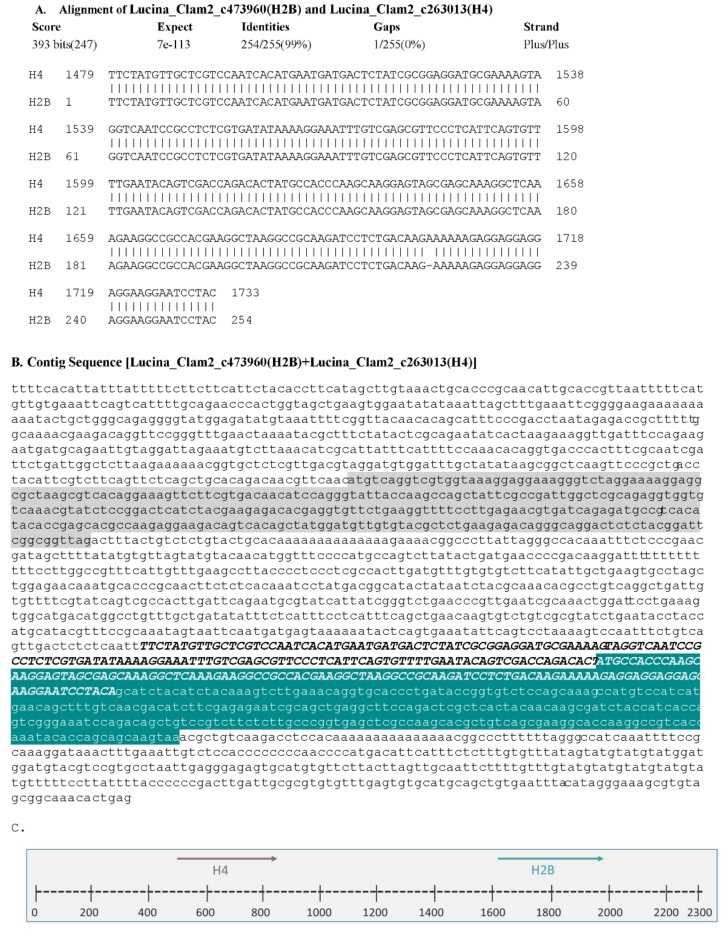
The H4 gene precedes the H2B gene in the *Lucina pectinata genome*. (**A**) BLASTN analysis between sequences containing the H4 and H2B genes obtained from the draft genome show an overlap of 255 bp, suggesting these two sequences can form a larger contig. (**B**) The contig containing the H4 and H2B genes resulted in a 2337 bp long sequence. The H4 coding region is highlighted in gray and the H2B coding sequence is highlighted in teal. The common overlapping region of 255 bp is indicated in bold/italic uppercase letters. (**C**) Schematic representation of the H4 (gray colored arrow) and H2B (teal colored arrow) genes localization, arrows indicate the direction of transcription (5′→3′).

With the draft genome assembly, we were able to identify at least one histone of each group, however, we selected only those sequences that contained a complete ORF, several sequences that had partial coding sequences were not considered (Figure 4, Figure 5, Figure 6, Figure 7 and Figure 8). Hence, despite the fact we have a large number of contigs, the draft genome assembly is adequate for the identification of relatively short genes. Two contigs had overlapping sequences, which when joined resulted in a larger contig harboring both an H4 and an H2B gene (Figure 9), which suggests that the *L. pectinata* histone genes may be organized in the typical clusters seen in other organisms, preserving the common gene order as well [22]. The sequences obtained were submitted to Genbank (Histone_1.A: MH760403; Histone_1.B: MH760404; Histone_2B MH760405; Histone_2A.W MH890684, Histone_2A.X MH760406; Histone_2A.Y MH760407; Histone_2A.Z MH890685, Histone_3 MH760408; Histone_4 MH760409).

The histone RNA sequences retrieved from the transcriptome assembly were partially confirmed by RT-PCR. All histone mRNAs had the typical palindromic sequence (underlined in Figure 4, Figure 5, Figure 6, Figure 7 and Figure 8) that is capable of forming a stem-loop structure, typical of replication-dependent (RD) histone genes, close to the stop codon. The conserved central domain found in histone H1 proteins was also found in the two H1 variants we identified in our study. At the nucleotide level, Purine-rich elements (in italics in Figure 4, Figure 5, Figure 6, Figure 7 and Figure 8) were only detected close to the palindromic sequence in the H1_A, H2A_X, H3 and H4, but their position was either slightly more distant to the palindromic sequence (H1_A, H2A_X and H3) or immediately preceding it (H4). The lack of these conserved sequences in the 3′end of the H1_B gene suggest it may belong to the replication-independent (RI) H1 isoforms, but we did not detect a polyadenylation signal, as seen in the mussel *Mytilus galloprovincialis* histone H1 “orphon” genes [23]. Hence, the histone H1_B gene expression pattern warrants further study to determine if its unusual 3′UTR has functional significance.

### 3.2. Multiple Sequence Alignment of Histone H1_A Variant Sequence Shows Conservation of Amino Acids Among Species

A BLAST search using the UniProtKB database with the H1_A from *L. pectinata* amino acid sequence as query was used to select sequences for alignment (Figure S1). The species selected include: mammals, mollusks, insects, fish, worms, reptiles and amphibians (Table S1). The multiple sequence alignment shows some gaps but most importantly shows regions where a large number of amino acids are conserved among the different species as shown in Figure S2. Among these amino acids the most conserved, which are shown in a darker shade of blue, are: lysine, valine, glycine, alanine, serine, tyrosine, methionine among others of less conservation (lightest shade of color). The most conserved amino acid is, as expected, lysine (K), a positively charge amino acid typically enriched in histones and involved in epigenetic histone modifications.

### 3.3. Phylogenetic Analysis of Lucina Pectinata Histone H1 Shows a Common Ancestor Among Other Histone H1 Proteins from Mollusks and Other Species

**Figure 10:**
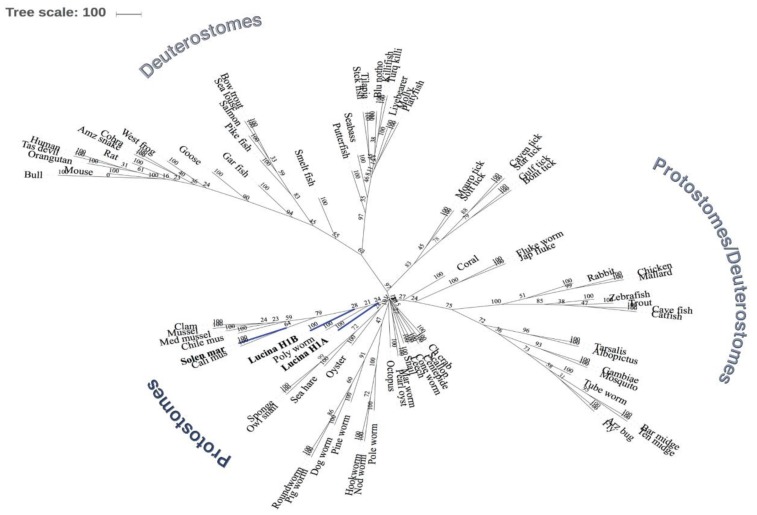
Phylogenetic Analysis between Histones H1 proteins of *Lucina pectinata* and Histone H1 protein from various species. An unrooted maximum likelihood (ProML) phylogenetic gene tree model was built from the CLUSTALW alignment. Numbers for nodes (interior of branches) represent bootstrap values based on 100 replicates. The *L. pectinata* query sequences (H1_A and H1_B) and the *S. marginatus* H1 protein sequence are shaded in blue. The legend provided represents the subtaxons of protostomes and deuterostomes.

We built a phylogenetic gene tree using the Maximum Likelihood Method (ProML) (PHYLIP) [19], and the results were visualized using the iTOL server [20]. As shown in Figure 11, our query sequences have a common ancestor that is closer in evolution to other marine organism, insects and birds. The results show that our query sequences are also related to the *S. marginatus* H1 protein, as expected, and to other bivalves or marine organisms (such as: sponges, mussels, other clams, oysters, octopus, crabs). *L. pectinata* H1_A and H1_B proteins are close in relationship with other protostome organisms (other mollusk, insects and worms). In comparison with studies of the razor clam *Solen marginatus* [1], another bivalve mollusk, our tree shows some similarities in evolutionary relationships between protostomes and deuterostomes as shown in Figure 10. Additionally, we can assume that the *L. pectinata* H1 proteins have a common ancestor not only with one another but also with *S. marginatus*, this relationship is represented in clades, which determine all the descendants of one common ancestor. Furthermore, comparing *L. pectinata’s* proteins with those from *S. marginatus*, H1_B from *L. pectinata* has a closer evolutionary relationship than H1_A to the *S. marginatus* H1. It is interesting that both H1_A and H1_B are both outgroups of their respective nodes. This finding suggests that H1_B is more evolved than H1_A, and also that H1_A has reached its highest evolutionary point in contrast to H1_B which kept on developing like the H1 from *S. marginatus*. As a summary of the results obtained for our phylogenetic analysis, our query proteins have an evolutionary relationship with other protostome organisms, but in contrast, they are farther apart in evolution from deuterostome organisms (including mammals). We can only assume that these proteins have evolved differently depending on the organism and also on the environment they are exposed to. Further studies of invertebrate hemoglobins are needed to better understand the evolution of histone H1, in addition to the evolution of histone H2A and its variants among species. 

## 4. Discussion

In order to validate the contigs of the genome assembly by identifying new proteins, we searched for genes that lacked introns and focused on the histone genes. The genome assembly contained too many contigs, which could be a consequence of several factors: repetitive sequences, polymorphisms, missing data and mistakes [24]. We found many repetitive elements located across *L. pectinata* genome [7]. From a computational perspective, sequence repeats have always presented technical challenges for sequence alignment and assembly programs, which, in turn, can produce biases and errors when interpreting results [25].

Since repetitive sequences are commonly found in introns rather than exons [26], we chose to search for genes coding for histone proteins, which are short, intron-less genes [27] and we expected that their sequence assembly would be easy and accurate. Histones are found in the somatic cells of all eukaryotic organisms, with the exception of some dinoflagellates [21,22]. In order to find the histone sequences in our genome assembly, we identified homologous sequences from another clam, *S. marginatus*. With these sequences we were able to retrieve at least one nucleotide sequence for each histone gene with relatively high identities at the nucleotide and predicted protein histone sequence levels for *L. pectinata* and, as expected, the conservation at protein level was higher than at the nucleotide level. The discontinuous SDS-PAGE analysis of histones isolated from the mantle tissue showed that there are additional polypeptides that could come from histone variants, which we found evidence of at the sequence level for the H1 and H2A genes, due to multiple copies of histone genes in the clam’s genome [22]. Alternatively, the multiple bands detected could be due to post- translational modifications or degradation or co-purification of abundant non-histone proteins. Histone variants are used to produce specialized nucleosomes, for example, the specialization imparted by H2A variants (H2A.X and H2A.Z), in the mussel *Mytilus galloprovincialis*, constitutes the earliest response to DNA damage [28]. Chromatin can be modified by the incorporation of histone variants, which would change local chromatin structure by promoting nucleosome subunit exchange to facilitate cellular processes such as transcription or during development [29]. Since our *L. pectinata* genome overall assembled size was estimated at 1.3 Gb and the estimated size of its genome is about 1.6 Gb, it is likely that we did not identify all of its histone gene sequences. Nevertheless, the sequence conservation at the nucleotide and protein levels showed that the clam histone H1 sequences have evolved more than their mammalian counterparts, which would be expected from an organism that lives in an extreme, sulfide-rich environment.

Our phylogenetic analysis is based on an unrooted tree which is an important step towards obtaining a representation of the evolutionary history of our selected histone H1 proteins. This representation shows us that the *L. pectinata*’s H1 proteins have an evolutionary relationship to other invertebrate organisms. As already shown, even though histone H1 proteins have conserved regions in their sequence, it is also the most variable of the histone proteins among species. This information can be seen in our phylogenetic analysis which demonstrates the relationship of the *L. pectinata* H1 proteins among different species. In our analysis, our query proteins fall closely in evolution to other H1 proteins of mollusk organisms (such as *S. marginatus*). In contrast, they are further apart in their evolutionary relationship to vertebrate species (such as mammals), other marine species (fish) and a variety of insects. Thus, even though histone H1 proteins have highly conserved regions that may not allow dissection of evolutionary relationships among other H1 proteins of a variety of species, when using the entire amino acid sequences, one may observe differences in evolutionary relationships between histone H1 proteins of various species. Future directions will include the construction of a rooted phylogenetic tree (from our unrooted tree) to define a clear view of the direction of evolution changes of histone H1 among various species and phylogenetic analyses of the histone H2A variants identified in our study.

## 5. Conclusions

In conclusion, we were able to identify histone gene sequences of each of the five types of histones in the *Lucina pectinata* genome assembly generated by semiconductor sequencing. These sequences were amplified by RT-PCR and sequenced. All of them had an identical match with those sequences obtained from the genome assembly. This suggests that, despite the fact that the assembly has many contigs, the clam genome and transcriptome assemblies are adequate for the identification of relatively short genes. In order to obtain a better genome assembly, new mate-paired libraries would be required to resolve regions with repetitive sequences. These studies will allow us to perform future studies on the epigenetic changes that may arise in chromatin proteins like the histones upon exposure to extreme environments.

We have been able to characterize histone genes and several gene variants from a clam that can withstand the extreme sulfide-rich environments of tropical mangroves and learn from the phylogenetic analysis how environmental influences have caused the most variable histone genes to develop gene variants that showed intra-species variability. In working with this clam, we had to overcome problems associated with a high repetitive sequence content as well as the nature of the sample, which often proved challenging for obtaining DNA or RNA adequate for molecular analyses. Nevertheless, we have produced two valuable resources for the academic community which can be mined to study gene and genome structure and evolution.

## Figures and Tables

**Table 1:** Primers used for the amplification of histone-like sequences from *Lucina pectinata* RNA by RT-PCR.

Target Sequence	Forward Primer	Reverse Primer
Histone H1_A	5′-GTCACGGCTCAATCCATAGT-3′	5′-GTGTGATAAATGAGAAGGTGATAGAAG-3′
Histone H2A	5′-GGCGATGCCTCATATTCTTT-3′	5′-AAAAAGGGCCGTTGAAAGTT-3′
Histone H2B	5′-CTTTGCGGAAAATTTGATGG-3′	5′-GCGTTCCCTCATTCAGTGTT-3′
Histone H3	5′-AAAAAGGGCCGTTATAGTGAG-3′	5′-ACCAATCAGCGTTGTTTTCC-3′
Histone H4	5′-AATAAGGGCCGTTTTCTTTTT-3′	5′-CGGGTTCTTCAGTTCTCAGC-3′

**Table 2:** Histone Nucleotide sequences identified from the BLASTN searches using the *Lucina pectinata* partial Histone H3 sequence and histone nucleotide sequences from the clam *Solen marginatus* as queries against the *L. pectinata* genome assembly.

Query Seq-id	Subject Seq-id	% Identity	Alignment Length	Number of Mismatches	# of Gaps	Start Query	End Query	Start subject	End Subject	E-value	Bit score
L.pectinataH3	Lucina_Clam2_rep_c512804	99.7	328	1	0	1	328	139	466	9.00E-173	589
S.margina-tus.H3	Lucina_Clam2_rep_c512804	82.75	458	74	2	200	652	115	572	2.00E-135	466
S.margina-tus.H1	Lucina_Clam2_c212178	68.98	432	92	12	251	670	1899	1498	1.00E-42	159
S.margina-tus.H1	Lucina_Clam2_rep_c490797	68.98	432	92	12	251	670	520	921	1.00E-42	159
S.margina-tus.H1	Lucina_Clam2_rep_c490798	68.98	432	92	12	251	670	420	821	5.00E-43	159
S.margina-tus.H2B	Lucina_Clam2_c473960	76.92	429	89	5	171	593	690	266	9.00E-90	315
S.margina-tus.H3	Lucina_Clam2_rep_c512805	81.37	424	73	3	234	652	1	423	9.00E-116	401
S.margina-tus.H2A	Lucina_Clam2_c411227	76.47	391	88	2	146	532	694	1084	6.00E-81	286
S.margina-tus.H2A	Lucina_Clam2_c263014	76.67	390	88	1	146	532	52	441	1.00E-82	291
S.margina-tus.H2A	Lucina_Clam2_c46738	68.51	362	107	2	151	507	241	600	6.00E-36	136
S.margina-tus.H2A	Lucina_Clam2_c75853	67.04	361	105	5	151	505	857	505	8.00E-27	105
S.margina-tus.H2B	Lucina_Clam2_c411226	79.2	327	65	1	171	497	417	740	4.00E-80	282
S.margina-tus.H4	Lucina_Clam2_c263013	79.62	319	65	0	147	465	555	873	9.00E-80	282
S.margina-tus.H4	Lucina_Clam2_rep_c482374	79.62	319	65	0	147	465	5	323	7.00E-80	282
S.margina-tus.H4	Lucina_Clam2_rep_c482375	79.31	319	65	1	147	465	46	363	4.00E-78	275
S.margina-tus.H1	Lucina_Clam2_c353214	69.18	305	81	7	302	601	317	613	3.00E-27	107
Solen.marginatus.H2A	Lucina_Clam2_rep_c481900	76.6	282	65	1	146	427	281	1	1.00E-57	208
Solen.marginatus.H3	Lucina_Clam2_rep_c482111	83.27	275	46	0	200	474	327	601	3.00E-82	289
Solen.marginatus.H3	Lucina_Clam2_c3893	74.35	269	65	2	200	466	1111	845	5.00E-45	168
Solen.marginatus.H2A	Lucina_Clam2_c155385	72.08	265	73	1	143	407	722	985	1.00E-37	141
Solen.marginatus.H1	Lucina_Clam2_rep_c482183	69.61	204	35	7	473	670	11	193	2.00E-19	82.4

**Table 3:** Summary of properties of *Lucina pectinata* histone proteins.

Type of Histone	Protein Size^1^	pI^2^	MW^3^	*Solen Marginatus* Protein Size^3^	*Solen Marginatus* Protein pI^2^	*Solen Marginatus* Protein MW^3^
H1_A	186	10.94	19,658	190	11.18	19,918
H1_B	179	11.29	18,750			
H3	136	11.27	15,388	136	11.47	15,521
H4	103	11.36	11,367	103	11.36	11,441
H2A_W	135	10.12	14,687	125	10.90	13,435
H2A_X	126	10.90	13,415			
H2A_Y	127	10.58	13,377			
H2A_Z	125	10.62	13,379			
H2B	125	10.52	13,855	124	10.71	13,837

^1^amino acid length of polypeptide; ^2^pI, isoelectric point; ^3^MW, molecular weight;

## Supplementary Materials

The following are available online at http://www.mdpi.com/1660-4601/15/10/2170/s1, Figure S1: Multiple Sequence Alignment generated using CLUSTALW; Figure S2: Multiple sequence alignment of the conserved central globular domain of histone H1s from 80 different species; Table S1: Complete List of H1 Protein Sequences Used for the Multiple Sequence and Phylogenetic Analyses.

## References

[1] González-Romero R., Ausió J., Méndez J., Eirín-López J. (2009). Histone genes of the razor clam *Solen marginatus* unveil new aspects of linker histone evolution in protostomes. Genome.

[2] Olins A.L., Olins D.E. (1974). Spheroid chromatin units (v bodies). Science.

[3] Fyodorov D.V., Zhou B.R., Skoultchi A.I., Bai Y. (2018). Emerging roles of linker histones in regulating chromatin structure and function. Nat. Rev. Mol. Cell Biol..

[4] Ponte I., Vila R., Suau P. (2003). Sequence complexity of histone H1 subtypes. Mol. Biol. Evol..

[5] Thoma F., Koller T. (1997). Influence of histone H1 on chromatin structure. Cell.

[6] Szerlong H.J., Hansen J.C. (2011). Nucleosome distribution and linker DNA: Connecting nuclear function to dynamic chromatin structure. Biochem. Cell Biol..

[7] Eirín-López J., Fernanda M., González-Tizón A., Martínez A., Méndez J. (2004). Birth-and-Death Evolution with Strong Purifying Selection in the Histone H1 Multigene Family and the Origin of *orphon* H1 Genes. Mol. Biol. Evol..

[8] Montes-Rodríguez I.M., Cadilla C.L., González-Méndez R., López-Garriga J., Ropelewski A. De Novo Assembly of Lucina pectinata Genome using Ion Torrent Reads. Proceedings of the Practice and Experience in Advanced Research Computing 2017 Proceedings (PEARC17).

[9] Chomczynski P., Sacchi N. (1987). Single-Step Method of RNA Isolation by Acid Guanidinium Extraction. Anal. Biochem..

[10] Krebs S., Fischaleck M., Blum H. (2009). A simple and loss-free method to remove TRIzol contaminations from minute RNA samples. Anal. Biochem..

[11] Grabherr M.G., Haas B.J., Yassour M., Levin J.Z., Thompson D.A., Amit I., Adiconis X., Fan L., Raychowdhury R., Zeng Q. (2011). Full-length transcriptome assembly from RNA-Seq data without a reference genome. Nat. Biotechnol..

[12] Altschul S.F., Gish W., Miller W., Myers E.W., Lipman D.J. (1990). Basic Local Alignment Search Tool. J. Mol. Biol..

[13] Altschul S.F., Madden T.L., Schäffer A.A., Zhang J., Zhang Z., Miller W., Lipman D.J. (1997). Gapped BLAST and PSI-BLAST: A new generation of protein database search programs. Nucleic Acids Res..

[14] Johnson M., Zaretskaya I., Raytselis Y., Merezhuk Y., McGinnis S., Madden T.L. (2008). NCBI BLAST: A better web interface. Nucleic Acids Res..

[15] Wheeler D.L., Church D.M., Federhen S., Lash A.E., Madden T.L., Pontius J.U., Schuler G.D., Schriml L.M., Sequeira E., Tatusova T.A. (2003). Database resources of the National Center for Biotechnology. Nucleic Acids Res..

[16] Untergasser A., Nijveen H., Rao X., Bisseling T., Geurts R., Leunissen J.A.M. Primer3Plus, an enhanced web interface to Primer3. Nucleic Acids Research.

[17] Thomas J.O., Kornberg R.D. (1975). An octamer of histones in chromatin and free in solution. Proc. Natl. Acad. Sci. USA.

[18] Waterhouse A.M., Procter J.B., Martin D.M.A., Clamp M., Barton G.J. (2009). Jalview Version 2—A multiple sequence alignment editor and analysis workbench. Bioinformatics.

[19] Felsenstein J. (1996). Inferring phylogenies from protein sequences by parsimony, distance, and likelihood methods. Methods Enzymol..

[20] Letunic I., Bork P. (2016). Interactive tree of life (iTOL) v3: An online tool for the display and annotation of phylogenetic and other trees. Nucleic Acids Res..

[21] Van Holde K.A., Richi A. (1988). Chapter 4: The Proteins of Chromatin. I. Histones. Chromatin.

[22] Maxson R., Cohn R., Kedes L., Mohun T. (1983). Expression and Organization of Histone Genes. Ann. Rev. Genet..

[23] Eirin-López J.M., Ruiz M.F., Gonález-Tizón A.M., Martínez A., Ausió J., Sánchez L., Méndez J. (2005). Common evolutionary origin and birth-and-death process in the replication-independent histone H1 isoforms from vertebrate and invertebrate genomes. J. Mol. Evol..

[24] Baker M. (2012). De novo genome assembly: What every biologist should know. Nat. Methods.

[25] Treangen T.J., Salzberg S.L. (2013). Repetitive DNA and next-generation sequencing: Computational challenges and solutions. Nat. Rev. Genet..

[26] Oki N., Yano K., Okumoto Y., Tsukiyama T., Teraishi M., Tanisaka T. (2008). A genome-wide view of miniature inverted-repeat transposable elements (MITEs) in rice, *Oryza sativa* ssp. japonica. Genes Genet. Syst..

[27] Pandey N.B., Chodchoy N., Liu T.J., Marzluff W.F. (1990). Introns in histone genes alter the distribution of 3′ ends. Nucleic Acids Res..

[28] González-Romero R., Rivera-Casas C., Frehlick L.J., Méndez J., Ausió J., Eirín-López J.M. (2012). Histone H2A (H2A.X and H2A.Z) variants in molluscs: Molecular characterization and potential implications for chromatin dynamics. PLoS ONE.

[29] Mariño-Ramírez L., Kann M.G., Shoemaker B.A., Landsman D. (2005). Histone structure and nucleosome stability. Expert Rev. Proteom..

[30] Towns J., Cockerill T., Dahan M., Foster I., Gaither K., Grimshaw A., Hazlewood V., Lathrop S., Lifka D., Peterson G.D. (2014). XSEDE: Accelerating scientific discovery. Comput. Sci. Eng..

[31] Nystrom N., Welling J., Blood P., Goh E.L., Vetter J.S. (2013). Blacklight: Coherent Shared Memory for Enabling Science. Contemporary High Performance Computing: From Petascale toward Exascale, CRC 2013.

[32] Nystrom N.A., Levine M.J., Roskies R.Z., Scott J. Bridges: A uniquely flexible HPC resource for new communities and data analytics. Proceedings of the 2015 XSEDE Conference, Scientific Advancements Enabled by Enhanced Cyberinfrastructure.

