# Supplementary material for: Characterization of Histone Genes from the Bivalve Lucina Pectinata
Source: Int J Environ Res Public Health. 2018 Oct 2;15(10):2170. doi: 10.3390/ijerph15102170 (PMC6210712; doi:10.3390/ijerph15102170)
Supplement: Supplementary file 1 [file ijerph-15-02170-s001.pdf]

International Journal of
Environmental Research and Public Health

MDPI

Supplementary Materials:

Figure S1. ClustalW alignment of 80 H1 protein amino acid sequences.

```
Mouse           ------------------------------------------------------------
Rat             ------------------------------------------------------------
Bull            ------------------------------------------------------------
Human           ------------------------------------------------------------
Orangutan       ------------------------------------------------------------
Amz_snake       ------------------------------------------------------------
Cobra           ------------------------------------------------------------
Tas_devil       ------------------------------------------------------------
West_frog       ------------------------------------------------------------
Goose           ------------------------------------------------------------
Salmon          ------------------------------------------------------------
Sea_louse       ------------------------------------------------------------
Bow_trout       ------------------------------------------------------------
Pike_fish       ------------------------------------------------------------
Smelt_fish      ------------------------------------------------------------
Gar_fish        ------------------------------------------------------------
Killifish       ------------------------------------------------------------
Turq_killi      ------------------------------------------------------------
Blu_notho       ------------------------------------------------------------
Seabass         ------------------------------------------------------------
Tilapia         ------------------------------------------------------------
Platyfish       ------------------------------------------------------------
Molly           ------------------------------------------------------------
Livebearer      ------------------------------------------------------------
Stck_fish       ------------------------------------------------------------
Putterfish      ------------------------------------------------------------
Cone_worm       ------------------------------------------------------------
Octopus         ------------------------------------------------------------
Jap_fluke       ------------------------------------------------------------
Fluke_worm      ------------------------------------------------------------
Ch_crab         ------------------------------------------------------------
Coral           MSGRGKGKAKGTKSKSRSSRAGLQFPVGRIHRLLRKGNYAERVGAGAPVYLAAVLEYLSA
Mar_worm        ------------------------------------------------------------
Lucina_H1B      ------------------------------------------------------------
Poly_worm       ------------------------------------------------------------
Cayen_tick      -----------------------------------------------------------L
Star_tick       ------------------------------------------------------------
Gulf_tick       --------------------------------------------------SLFRSPTGSD
Bont_tick       ------------------------------------------------------------
Soft_tick       ------------------------------------------------------------
Mouro_tick      ------------------------------------------------------------
Clam            ------------------------------------------------------------
Med_mussel      ------------------------------------------------------------
Mussel          ------------------------------------------------------------
Chile_mus       ------------------------------------------------------------
Cali_mus        ------------------------------------------------------------
Solen_mar       ------------------------------------------------------------
Lucina_H1A      ------------------------------------------------------------
Leech           ------------------------------------------------------------
Sponge          -----------------------------------------------------------M
Owl_snail       ------------------------------------------------------------
Sea_hare        ------------------------------------------------------------
Oyster          ------------------------------------------------------------
Snail           ------------------------------------------------------------
Pearl_oyst      ------------------------------------------------------------
Scallop         ------------------------------------------------------------
Centepide       ------------------------------------------------------------
Pig_worm        ------------------------------------------------------------
Roundworm       ------------------------------------------------------------
Dog_worm        ------------------------------------------------------------
Pine_worm       ------------------------------------------------------------
Nod_worm        ------------------------------------------------------------
Hookworm        ------------------------------------------------------------
Pole_worm       ------------------------------------------------------------
Cave_fish       ------------------------------------------------------------
Catfish         ------------------------------------------------------------
Zebrafish       ------------------------------------------------------------
Trout           ------------------------------------------------------------
Chicken         ------------------------------------------------------------
Mallard         ------------------------------------------------------------
Rabbit          ------------------------------------------------------------
Bar_midge       ------------------------------------------------------------
Ten_midge       ------------------------------------------------------------
Fly             ------------------------------------------------------------
Arz_bug         ------------------------------------------------------------
Tarsalis        ------------------------------------------------------------
Albopictus      ------------------------------------------------------------
```

```
Gambiae         ------------------------------------------------------------
Mosquito        ------------------------------------------------------------
Tube_worm       ------------------------------------------------------------


Mouse           -----------------------------------------MTENSTSAPAA--------
Rat             -----------------------------------------MTENSTSTPAA--------
Bull            -----------------------------------------MTENSTSTPAA--------
Human           -----------------------------------------MTENSTSAPAA--------
Orangutan       -----------------------------------------MTENSTSAPAA--------
Amz_snake       -----------------------------------------MTENSTTAPAA--------
Cobra           -----------------------------------------MTENSTTAPAA--------
Tas_devil       -----------------------------------------MTEN--SAPAS--------
West_frog       -----------------------------------------MTENSAAAPAG--------
Goose           ----------------------------------------TDSPIPAPAPAA--------
Salmon          -----------------------------------------MAET-AAAPAP--------
Sea_louse       -----------------------------------------MAET-AAAPVP--------
Bow_trout       -----------------------------------------MAET-AAAPAP--------
Pike_fish       -----------------------------------------MAET-VAAPAP--------
Smelt_fish      -----------------------------------------MAET-AAAPAP--------
Gar_fish        -------------------------------------KTPVMAETAAAAPAQ--------
Killifish       -----------------------------------------MAET--SGAPA--------
Turq_killi      -----------------------------------------MAET--SGAPA--------
Blu_notho       -----------------------------------------MAET--SGAPA--------
Seabass         -----------------------------------------MAET--SAAPA--------
Tilapia         -----------------------------------------MTET--STAPA--------
Platyfish       -----------------------------------------MAET--SAAPA--------
Molly           -------------------------EFLINFIFKRRIEEVTMAET--SATPA--------
Livebearer      -----------------------------------------MAET--SAAPA--------
Stck_fish       ---------------------------------------VTMAET--STAPA--------
Putterfish      -----------------------------------------MAET--SAAPA--------
Cone_worm       -----------------------------------------MADAIKS------------
Octopus         -----------------------------------------MAETTEQK-----------
Jap_fluke       -----------------------------------------MTVSAISGGAIAAAP----
Fluke_worm      -----------------------------------------MTVSGVSGGSLAAAP----
Ch_crab         ---------------------------------------------MADSAAA--------
Coral           EILELAGNAARDNKKTRIIPRHLQLAVRNDEELNKLLAGVTIAQGVHNMSEV--------
Mar_worm        -----------------------------------------MSEAVSSAAKK--------
Lucina_H1B      -----------------------------------------MS-AAPVKAKK--------
Poly_worm       -----------------------------------------MSDVAAPKAKK--------
Cayen_tick      LFSSLRCVLVIRHPFLVR---CSSTDTLPAPVMSDVEVKPAAPATPKPKGRKP-------
Star_tick       -----------------------------------AEVKPAVPPTPKAKGRKS-------
Gulf_tick       VLRSARCVPVTSCHFLQFPSAVPGTDTLPTTVMSDAEVKPAAAATPKAKGRKS-------
Bont_tick       --------------------------------MSDAEVKPAAAATPKAKGRKS-------
Soft_tick       --------------------------------MSETEAA-KAPATPKTKGRK--------
Mouro_tick      --SAFVRVLHVSS---------------PFTMSAEAEAA-KPAASPKGKGRK--------
Clam            --------------------------------MAD---ATAAPAVAPAKSP---------
Med_mussel      --------------------------------MAD---ATAAPAVAPAKSP---------
Mussel          --------------------------------MAD---ATAAPAVAPAKSP---------
Chile_mus       --------------------------------MAD---AIAAPAPAPAKSP---------
Cali_mus        --------------------------------MAD---AKAAPAAAPANSP---------
Solen_mar       --------------------------------MAD---AKAAPAAAPANSP---------
Lucina_H1A      --------------------------------MAD---TAVAPAAAATPKPA--------
Leech           --------------------------------MSDE-QVEQAPATAPVEEKKKAP-----
Sponge          TLEIRICHEITNTRFISSQNQKKNDVRKKQSKMTDA------AVAPVHKSP---------
Owl_snail       --------------------------------MTDA------AVAPVHKSP---------
Sea_hare        --------------------------------MSDAP----AAVAQTKTTPA--------
Oyster          --------------------------------MSDA------VVATP--KPA--------
Snail           --------------------------------MEDSTNATPAAATAPAVTTPAKA-----
Pearl_oyst      --------------------------------MSDAEVAAPAEATP--VKKGAKT-----
Scallop         --------------------------------MSDSEEVTTPAAEDAPAPAAAP------
Centepide       --------------------------------MS--ENATAPTTGRGRTKTAA-------
Pig_worm        --------------------------------MS---TEVAAPAPPAATETKK--P----
Roundworm       --------------------------------MS---TEVAAPAPPAATETKK--P----
Dog_worm        --------------------------------MS---TEVATAGSPATAEAKK--P----
Pine_worm       --------------------------------MSDSAAPVAAPASPKAPASPK--A----
Nod_worm        --------------------------------MS----DAAVAAPTPTAPAPV--T----
Hookworm        --------------------------------MS----DAVVAPPTPTAPATA--T----
Pole_worm       --------------------------------MS----DAAPATSP---------V----
Cave_fish       --------------------------------MAEIAPA--ASAPA---KAPKKK-----
Catfish         --------------------------------MAEVAPAP-AVAPA---KAPKKK-----
Zebrafish       --------------------------------MAETAPAP-AASPA---KAPKKK-----
Trout           ---------------------------------AEVAPAPAAAAPA---KAPKKK-----
Chicken         --------------------------------MSETAPVAAPAVSAPGAKAAAKKP----
Mallard         --------------------------------MSETAPVAAPAVSAPGAKAAGKKP----
Rabbit          --------------------------------MSETAP-AAPAAPAPAEKTPVKKK----
Bar_midge       --------------------------------MSDPAPEVAP---AAPVASPAK---AKK
Ten_midge       --------------------------------MSDPAQEVEAPVEAAPVASSPK---GKK
Fly             --------------------------------MSDSAVATSASPVIAQAASGEKKVSTKK
Arz_bug         --------------------------------MADTAANAAAP-----AASPKK---GKA
Tarsalis        --------------------------------MTETATEVVEAAPAAAASP-AKA--TKK
Albopictus      -----------------------------------TTTEVAAAAP-AAASP-AKT--PKK
Gambiae         -------------------------------MADTAATEAPTAAAAAAPAPAAKS--PKK
Mosquito        -------------------------------MADTAATEAPTAAAAAAPAPAAKS--PKK
Tube_worm       --------------------------------MADSAPATAAP------ATPTK---AKK


Mouse           ------------KPKRAKASKKS--------TDHPKYSDMIVAAIQAEKNRAGSSRQSIQ
Rat             ------------KPKRAKAAKKS--------TDHPKYSDMIVAAIQAEKNRAGSSRQSIQ
```

```
Bull            ------------KPKRAKASKKS--------TDHPKYSDMIVAAIQAEKNRAGSSRQSIQ
Human           ------------KPKRAKASKKS--------TDHPKYSDMIVAAIQAEKNRAGSSRQSIQ
Orangutan       ------------KPKRAKASKKS--------TDHPKYSDMVVAAIQAEKNRAGSSRQSIQ
Amz_snake       ------------KPKRAKAAKKS--------TDHPKYSDMIVAAIQAEKSRAGSSRQSIQ
Cobra           ------------KPKRAKAAKKS--------TDHPKYSDMIVAAIQAEKSRAGSSRQSIQ
Tas_devil       ------------KPKKAKASKKS--------TDHPKYSDMIVAAIQAEKNRAGSSRQSIQ
West_frog       ------------KPKRSKASKKA--------TDHPKYSDMILAAVQAEKSRSGSSRQSIQ
Goose           ------------KPKRARAPRKP--------ASHPTYSEMIAAAIRADKSRGGSSRQSIQ
Salmon          ------------KAKKAKAPKKP--------ASHPKYSDMIKAAVHADKSRGGASRQSVQ
Sea_louse       ------------KAKKAKAPKKP--------ASHPKYSDMIKAAVHADKSRGGASRQSVQ
Bow_trout       ------------KAKKAKAPKKP--------ASHPKYSDMIKAAVHADKSRGGASRQSVQ
Pike_fish       ------------KAKKAKAPKKP--------ASHPKYSDMIKAAVQADKSRGGASRQSVQ
Smelt_fish      ------------KAKKAKTPKKT--------ASHPKYSDMIKTAIVADKSRGGASRQSIQ
Gar_fish        ------------KAKKAKGPKKA--------STHPKYSDMIEAAVHAEKSRGGASRQSIQ
Killifish       ------------KAKKASKPKKP--------ASHPKYSDMIKAAIVHDASRSGASRQSIQ
Turq_killi      ------------KAKKASKPKKP--------ASHPKYSDMIKAAIVHDASRSGASRQSIQ
Blu_notho       ------------KAKKASKPKKP--------ASHPKYSDMIKAAIVHDASRSGASRQSIQ
Seabass         ------------KAKKASKPKKP--------ASHPKYSDMIKAAIVHDASRSGASRQSIQ
Tilapia         ------------KAKKASKPKKP--------ASHPKYSDMIKAAIVHDASRSGASRQSIQ
Platyfish       ------------KAKRASKPKKP--------TSHPKYSDMIKAAIVHDASRNGASRQSIQ
Molly           ------------KAKRASKPKKP--------ASHPKYSDMIKAAIVHDASRSGASRQSIQ
Livebearer      ------------KAKRASKPKKP--------ASHPKYSDMIKAAIVNDASRSGASRQSIQ
Stck_fish       ------------KAKKAAKPKKA--------ASHPKYSEMIRAAIAHDASRSGASRQSIQ
Putterfish      ------------KAKKSSKPKKP--------ASHPKYSEMIKAAIVHDANRNGASRQSIQ
Cone_worm       ---------PTKKVAAAAKPKKV--------STHPKYSEMVRAAIEALKERGGSSRQAIL
Octopus         ---------AAATTKKAAKAKKA--------SRYPAYNTMITEAITALAEKGGSSRQAIS
Jap_fluke       ---------LSGGTKKSTKSKKTP-------SAHPPYGDMIKKALKELKERGGSSRQAIA
Fluke_worm      ---------STAGVRKSAKSKKAP-------SIHPPYGDMIKKALKELKERGGSSRQAIA
Ch_crab         ----------APAPKKAKKGAAKK-------TSHPKYSAMIAAAITSLKERGGSSRQAIL
Coral           ----------KPSPKKKPAAPKKP-------ADHPPYLDMIKAAIVALKERNGSSRQAIE
Mar_worm        ----------KPAAKKSKKGS----------GDHPAYKDMVVAAVAALKEKGGSSRQAIL
Lucina_H1B      ----------------AAKPKKP--------STHPKYSEMIAKAITSLKERGGSSRQAIL
Poly_worm       ----------------AAKPKKP--------AAHPKYSEMVQAAVTSLKERGGSSRQAIL
Cayen_tick      ------------AAGGPKKAKKP--------ASHPKYTEMVKKSIEALKERGGSSRQAIH
Star_tick       ------------TAGAPKKVKKP--------ASHPKYTEMVKKSIEALKERGGSSRQAIH
Gulf_tick       ------------AAGGAKKAKKP--------ASHPKYTEMVKKSIEALKERGGSSRQAIH
Bont_tick       ------------AAGSAKKAKKP--------ASHPKYTEMVKKSIEALKERGGSSRQAIH
Soft_tick       ------------SAG-AKKDKKP--------ANHPKYTDMVKKAVAALKERGGSSRQAIH
Mouro_tick      ------------SAGAAKKAKKP--------ANHPKYTDMVKSAIVDLKERGGSSRQAIH
Clam            ------------KKKAAAKPKKP--------SAHPKYSEMIGKAIAALKERGGSSRQAIL
Med_mussel      ------------KKKAAAKPKKP--------SAHPKYSEMIGKAIAALKERGGSSRQAIL
Mussel          ------------KKKAAARPKKP--------SAHPKYSEMIGKAIAALKERGGSSRQAIL
Chile_mus       ------------KKKAAAKPKKP--------SAHPKYSEMIGKAIAALKERGGSSRQAIL
Cali_mus        ------------KKKAAAKPKKP--------SAHPKYSEMIGKAIAALKERGGSSRQAIL
Solen_mar       ------------KKKAAAKRKKP--------SAHPKYSEMIGKAIAALKERGGSSRQAIL
Lucina_H1A      ------------KKKAAAKPKKP--------SAHPKYSEMIQAAVSNLKERGGSSRQAIL
Leech           ------------KKKAAPKAKKV--------ADHPKYAEMIKDALNALKERGGSSRQAIL
Sponge          ------------KKKTSAKPKVP--------AAHPKYVDMIKAAVASLKERGGSSRQAIL
Owl_snail       ------------KKKTSAKPKVP--------AAHPKYVDMIKAAVASLKERXGSSRQAIL
Sea_hare        ------------KKRAQGKPKAP--------PAHPQYKIMVVAAISSLKERGGSSRQAIL
Oyster          ------------KAKKAAKPKKP--------ASHPKYSDMIKAALTALKEKGGSSRQAIL
Snail           -----------QKKKAAAKPKSK------KPANHPKYCDMIKAAITNLKERGGSSRQAIL
Pearl_oyst      -----------AKTATKKKSKNKG-----PPKDHPMFKDMIKDAITALKERGGSSRQAIL
Scallop         ------------AEPAAAKKKKPSKSKPKKPSTHPKFRDMAVEAIGALKERSGSSRQAIL
Centepide       ---------------AAAKPKK-----PKTPASHPKYLEMSVAAINALKERNGSSRQAIL
Pig_worm        ---------AAKKTKAAGKPKA-----PKVPASHPAYGDMVKAAVVALKEKKGSSRAAIL
Roundworm       ---------AAKKTKAAGKPKA-----PKVPASHPAYGDMVKAAVVALKEKKGSSRAAIL
Dog_worm        ---------AAKKGRGAGRPKT-----PKTPASHPAYGDMVKAAVVALKEKKGSSRAAIL
Pine_worm       ---------SPKAKKVAKAPKAKAAAKPKTPANHPTYIDMIKKAITELKEKKGSSRAAIL
Nod_worm        ---------PVKKTKAA-KPKGEK--KAKATPSHPVYGAMIKAAIKELKDRKGASKQAIL
Hookworm        ---------STKKVKPS-KPKGEK--KAKVAPSHPVYGAMIKAAIKELKDRKGASKQAIF
Pole_worm       ---------PAKKTKAS-KPKGEK--KPKTAPSHPVYSAMIKAALKELKDRKGASKQAIL
Cave_fish       -----------------AAARPK--------KAGPSVGEMIVKAVSASKERSGVSLAALK
Catfish         -----------------AASRAK--------KAGPSVGDLIVKAVSSSKERSGVSLAAVK
Zebrafish       -----------------AASKPK--------KAGPNVRDLIVKTVTASKERNGVSLAALK
Trout           -----------------AAAKPK--------KSGPAVGELAGKAVAASKERSGVSLAALK
Chicken         ------------KKAAGGAKPRK--------PAGPSVTELITKAVSASKERKGLSLAALK
Mallard         ------------KKAAGGSKARK--------PAGPSVTELITKAVAASKERKGLSLAALK
Rabbit          ------------ARKSAGAAKRK--------ASGPPVSELITKAVAASKERSGVSLAALK
Bar_midge       EK--------KPKSDKPKKPKAP--------RTHPPVSEMVVNAIKTLKERGGSSLQAIK
Ten_midge       EKAPKAPKAEKPKSDKPKKPKAA--------PTHPPVSEMVVNAITTLKERGGSSLIAIK
Fly             AA---------ATPKSKKSTAAP--------PSHPPTQQMVDASIKNLKERGGSSLLAIK
Arz_bug         AA---------AAAAASKKPRVK--------PAHPPTSDMVNAAIKSLKERGGSSLQAIK
Tarsalis        PK---------AAKGDAKKPKKP--------STHPPVNEMVIAAIKTLKERNGSSLQAIK
Albopictus      TK---------AAKGDAKKPKKP--------ATHPPANEMVLAALKNLKERKGSSLQAVK
Gambiae         PK---------AAAG-PKKPKQP--------AAHPPINEMLLAAVKALNERNGSSLQAIK
Mosquito        PK---------AAAG-PKKPKQP--------AAHPPINEMMLAAVKALNERNGSSLQAIK
Tube_worm       AA-------------APKKPKVP--------AAHPPVAQMVNAAIAALKDRKGSSLAAIK
                                                  *    :   ::    .: * *  ::

Mouse           KYIKSHYKVG---ENADSQIKLSIK-RLVTTGVLKQTKGVGASGSFRLAKG---------
Rat             KYIKSHYKVG---ENADSQIKLSIK-RLVTTGVLKQTKGVGASGSFRLAKG---------
Bull            KYIKSHYKVG---ENADSQIKLSIK-RLVTTGVLKQTKGVGASGSFRLAKS---------
Human           KYIKSHYKVG---ENADSQIKLSIK-RLVTTGVLKQTKGVGASGSFRLAKS---------
Orangutan       KYIKSHYKVG---ENADSQIKLSIK-RLVTTGVLKQTKGVGASGSFRLAKS---------
Amz_snake       KYIKSHYKVG---ENADSQIKLSIK-RLVTTGVLKQTKGVGASGSFRLAKG---------
Cobra           KYIKSHYKVG---ENADSQIKLSIK-RLVTTGVLKQTKGVGASGSFRLAKS---------
Tas_devil       KYIKNHYKVG---ENADSQIKLSIK-RLVTNGVLKQTKGVGASGSFRLAKG---------
West_frog       KYIKNHYKVG---ENADSQIKLSIK-RLVTSGTLKQTKGVGASGSFRLAKA---------
```

```
Goose           KYVKSHYKVG---QHADLQIKLAIR-RLLTTGVLKQTKGVGASGSFRLAKG---------
Salmon          KYIKSHYKVG---DNADSQIKLSLK-RMVSEGVLRHTKGIGASGSFKLAKA---------
Sea_louse       KYIKSHYKVG---DNADSQIKLSLK-RMVSEGVLRHTKGIGASGSFKLAKA---------
Bow_trout       KYIKSHYKVG---DNADSQIKLSLK-RMVSEGVLRHTKGIGASGSFKLAKA---------
Pike_fish       KYIKSHYKVG---DNADSQIKLSLK-RMVSGGLLRHTKGIGASGSFKLAKA---------
Smelt_fish      KYIKGHYKVG---ENADSQIKLSLK-RLVNSGVLRHTKGIGASGSFKLAKA---------
Gar_fish        KYIKSHYKVG---DNADSQIKLSLK-RLVATGVLRHTKGVGASGSFKLAKA---------
Killifish       KYVRKTYKVG---DNADVQIKMALK-RLVASGMLRHTKGIGASGSFRLTKP---------
Turq_killi      KYVRKTYKVG---DNADVQIKMALK-RLVASGMLRHTKGIGASGSFRLTKP---------
Blu_notho       KYVRKTYKVG---DKADVQIKLALK-RLVASGMLRHTKGIGASGSFRLTKP---------
Seabass         KYVKKNYKVG---DNVDVQIKLALK-RLVASGMLRHTKGIGASGSFRLTKP---------
Tilapia         KYVRKNYKVG---DNADVQIKMALK-RLVAAGTLRHTKGIGASGSFRLTKP---------
Platyfish       KYVRKNYKVG---DNADVQIKMALK-RLVASGMLRHTKGIGASGSFRLTKP---------
Molly           KYVRKNYKVG---DNADVQIKMALK-RLVASGMLRHTKGIGASGSFRLTKP---------
Livebearer      KYVRKNYKVG---DNADVQIKMALK-RLVASGMLRHTKGIGASGSFRLTKP---------
Stck_fish       KYVRKNYKVG---ENADVQIKLALR-RLVAAGSLRHTRGIGASGSFRWAKP---------
Putterfish      KYVKKNYKVG---DNVDVQIKLALK-RLVESGMLRQTKGIGASGSFKLTKP---------
Cone_worm       RYIVLNYKVGEDERVVNQHLKVALR-TGVKSEMLKQSKGSGASGSFKLGD----------
Octopus         KYIVKQYKVSPDDKGAIAQLKLGLK-RGVRTGALKQSKGSGASGSFKLGS----------
Jap_fluke       KYIKANYKV--DER-AESHLRRALV-TGVKSGKIVHTKGIGASGSFKLADK---------
Fluke_worm      KYIKTNYKV--DDR-AESHLRRALV-TGVKSGKLVHTKGIGASGSFKLADK---------
Ch_crab         KHIIANNEVD--EKAAATQLKLALK-RGVASGALKQVKGAGASGSFRINKP---------
Coral           KYIKANYKVG--EVG--SHLKMALK-RGAASGKLLHTKGVGASGSFKVAKE---------
Mar_worm        KYVVAHYKVGN-EKVANVHLKISLR-RLVDGGQLKRVKGTGASGSFKIAQA---------
Lucina_H1B      KHIMANFNVGKDAAAVNAHLKMALK-RGVAKGALKQSKGTGASGSFRLG-----------
Poly_worm       KYIVKNFNVGTEEKTVNTHLKLALR-AGVAKGTLKQSKGTGAAGSFRMG-----------
Cayen_tick      KYIMSHFDVGKDSKVVNTHLKLALK-RAVQTGLLKRSKGTGASGSFRLA-----------
Star_tick       KHIMSHYDVGKDSKVVNTHLKLALK-RAVQSGLLKRSKGTGASGSFRLA-----------
Gulf_tick       KYIMGHYDVGKDTKVVNTHLKLALK-RAVQTGLLKHSKGTGASGSFRLA-----------
Bont_tick       KYIMGHYDVGKDTKVVNTHLKLALK-RAVQTGLLKHSKGTGASGSFRLA-----------
Soft_tick       KYIMANFDVGKDSKVVNTHLKLALK-RAVETGILKHAKGTGASGSFRLA-----------
Mouro_tick      KYIMANYDVGKDSKVVNTHLKQALK-RSVQTGALKRSKGTGASGSFRLA-----------
Clam            KYIMANFNVGKDAKSVNAHLKLALR-AGVKNNSLKQSKGTGASGSFRIG-----------
Med_mussel      KYIMANFNVGKDAKSVNAHLKLALR-AGVKNNSLKQSKGTGASGSFRIG-----------
Mussel          KYIMANFNVGKDAKSVNAHLKLALR-AGVKNNSLKQSKGTGASGSFRIG-----------
Chile_mus       KYIMANFNVGKDAKSVNAHLKLALR-AGVKNNSLKQSKGTGASGSFRIG-----------
Cali_mus        KYIMANFNVGKDAKSVNAHLKLALR-AGVKNNSLKQSKGTGASGSFRIG-----------
Solen_mar       KYIMANFNVGKDAKSVNAHLKLALR-AGVKNNRLKQSKGTGASGSFRIG-----------
Lucina_H1A      KYILANYKV--DEKQANSHLKLALR-AGVKNGSLKQSKGTGASGSFRLG-----------
Leech           KYIAQNYTLGNDEKVINSHLKMALK-AGVKNGSLKQSKGVGASGSFKIGNGD--------
Sponge          KYVMANYKVGTDLKAVNSRIKNALK-NGVKAGTLKQSKGTGAAGSFKLG-----------
Owl_snail       KYVMANYKVGTDLKAVNSRIKNALK-NGVKAGTLKQSKGTGAAGSFKLG-----------
Sea_hare        KYIIANYKVGDNPTAINARLKTALR-AGVKAETLKQSKGTGASGSFRLG-----------
Oyster          KYIIANNKV--DAEAASRHLRMALK-AGVKNGTLKQSKGTGASGSFKLG-----------
Snail           KYIMANYNVGKDEKVINSHLKMALR-AGAKNGNLKQSKGVGASGSFKLGA----------
Pearl_oyst      KHIVAHYNI-KDQKAANAHLKMALR-AGVKNGTLKQAKGTGASGSFKMG-----------
Scallop         KHIRANYSVDQASD--NNHLKMTLK-ACVKNGTLKQCKGTGASGSFKLG-----------
Centepide       KYITGHYSVGKDAKVVNTHLKMALK-RGVSSGVLKHAKGQGATGSFRVS-----------
Pig_worm        KYILQHYKVGDNLTAVNAHLRQALK-RGVSTGALKQTKGTGASGSFRLG-----------
Roundworm       KYILQHYKVGDNLTAVNAHLRQALK-RGVSTGALKQTKGTGASGSFRLG-----------
Dog_worm        KYILQHYKVGDNLTAVNAHLRLALK-RGVSTGALKQTKGTGASGSFRLG-----------
Pine_worm       KYILQHYKVGSNIVAINAHLRQGLK-RGVTTGALKQTKGVGASGSFRLA-----------
Nod_worm        KYITQKYKVGDNEKQINARLRLALK-KGVENGLLKQASGTGAAGRFRLA-----------
Hookworm        KFIIQKYKVGDNEKQINARLRLALK-KGVEKGLLKQASGTGAAGRFRLA-----------
Pole_worm       KFMTQKYKLGTNEKQINAHLRMALK-RGVSSGALTQPSGTGAAGRFRLA-----------
Cave_fish       KALAAG-GYDVE--KNNSRVKIAVK-SLVTKGTLVQTKGTGASGSFKLNKK--QAEAKKK
Catfish         KALAAG-GYDVE--KNNSRVKLAVK-GLVTKGTLVQTKGTGASGSFKLNRK--QTEAKK-
Zebrafish       KALSAG-GYDVE--KNNSRVKTAVK-ALVTNGTLAQTKGTGASGSFKLNKK--QAEPKK-
Trout           KSLAAG-GYDVE--KNNSRVKIAVK-SLVTKGTLVETKGTGASGSFKLNKK--AVEAKKP
Chicken         KALAAG-GYDVE--KNNSRIKLGLK-SLVSKGTLVQTKGTGASGSFKLNKKPGETKAKAT
Mallard         KALAAG-GYDVE--KNNSRIKLGLK-SLVGKGTLVQTKGTGASGSFKLNKKPGETKEKAT
Rabbit          KALAAA-GYDVE--KNNSRIKLGLK-SLVSKGTLVQTKGTGASGSFKLNKKAAAGEAKPK
Bar_midge       KFLVAQYKVDVE--KLAPFIKKYLKGGAVVKGELLQTKGKGASGSFKLPAAAKKEKVAKK
Ten_midge       KFVAAQYKVDVE--KLVPFIKKFLK-ASVAKGTLLQAKGKGASGSFKLPPAAKK---VDK
Fly             KYIGATYKCDAQ--KLAPFIKKYLK-NAVANGKLIQTKGKGASGSFKLSRSAKK---DPK
Arz_bug         KYIAANYKVDAE--KMAPFIKKYLK-AAVASGALVQPKGKGASGSFKLSGEGKT---TAV
Tarsalis        KYIAANYKCDVA--KLSTFIKKALK-TNVEKGKLVQTKGSGASGSFKIKAEAKKPAGEKK
Albopictus      KYIGANYKCDVA--KLSIFIRKALK-AGVEKGTFVQTKGSGASGSFKLK--DNKAAAEKK
Gambiae         KYVAANYKADVT--KLASFFKKALK-SGVASGKLVQTKGTGASGSFKLSAAAKKPVVEKK
Mosquito        KYVAANYKADVT--KLATFFKKALK-TAVANGKLVQTKGTGASGSFKLSAAAKKPAVEKK
Tube_worm       KYIAANYKCNID--RCTPFIRKYLK-QAVAAGKLVQTKGTGAAGSFKLSAAAKA----EK
                : :                 .:  :          :  .   * **:* *:

Mouse           -DEPKRS---VAFK--KTKKEVKKVAT---------PKKAAKPKKAA-SKAPSKKPKAT-
Rat             -DEPKRS---VAFK--KTKKEVKKVAT---------PKKAAKPKKAA-SKAPSKKPKAT-
Bull            -DEPKRS---VAFK--KTKKEVKKVAT---------PKKAAKPKKAA-SKAPSKKPKAT-
Human           -DEPKKS---VAFK--KTKKEIKKVAT---------PKKASKPKKAA-SKAPTKKPKAT-
Orangutan       -DEPKKS---VAFK--KTKKEIKKVAT---------PKKASKPKKAA-SKAPTKKPKAT-
Amz_snake       -DEPKK----APVK--KAKKEVKKATT---------PKKAAKPKKAV-AKSPAKKPKP--
Cobra           -DEPKK----APVK--KAKKEVKKATT---------PKKAAKPKKAA-AKSPAKKPKP--
Tas_devil       -DEPKK----PAVK--KAKKEVKKAAT---------PRKASKPKKAATAKTPAKKPKA--
West_frog       -DEGKK-----PAK--KPKKEIKKAAS---------PKKAAKPKKAAKSPAKAKKPKVAE
Goose           -DKAKRSP--AGRK--KKKKAARKSTS---------PKKAARPRKAR---SPAKKPKAA-
Salmon          -EDTKKAPKVKAVV--KAKKSPVKSAK---------PKKAAKPKKVAKSPAKKAKKAVAV
Sea_louse       -EDTKKAPKVKAVV--KAKKSPVKSAK---------PKKVAKPKKVAKSPAKAKKAKVAV
Bow_trout       -EDTKKAPKVKTVV--KAKKSPVKAAK---------PKKVAKPKKVAKSPVKAKKAKVAV
Pike_fish       -EDTKKAPKPKPVV--KAKKSPVKAAK---------PKKVAKPKKVVKSPAKAKKAKVAV
Smelt_fish      -EDAKKVSKPKPVV--KAKKP---AAK---------PKKVAKPKKVAKSPAKAKKPKPAA
Gar_fish        -DESKKAP--KPAE--KPKKKATKAAK---------PKKVAKPKKVTKSPVKAKKPKAVE
```

```
Killifish       -EDSKKP-AKAAAS--AKPKKVAKP-K---------PKKAAKPKKVPKTPEKPKKAAAK-
Turq_killi      -EDSKKP-AKAAAS--AKPKKVAKP-K---------PKKAAKPKKVPKTPEKPKKAAAK-
Blu_notho       -EDSKKP-AKAAAS--AKPKKVAKP-K---------PKKAAKPKKVPKTPEKPKKAAAK-
Seabass         -EDSKKP-TKAAAT--AKPKKAAKPSK---------PKKAAKPKKVPKTPEKPKKAAAK-
Tilapia         -EDSKKS-TKAAAVTAPKPKKAAKPAK---------PKKVAKPKKVAKTPEKPKKAAAK-
Platyfish       -EDSKKPPAKAPTA--AKPKKVAKP-K---------PKKAAKPKKVTKTPEKPKKAAVK-
Molly           -EDSKKPPAKAPAA--AKPKKVAKP-K---------PKKAAKPKKVTKTPEKPKKAAVK-
Livebearer      -EDSKKPPAKAPAA--AKPKKVAKP-K---------PKKAAKPKKVTKTPEKPKK-AVK-
Stck_fish       -EDDKKP-----VP--AKAKKAAKPSK---------PKKAAKPKKVAKTPEKPKKAAAK-
Putterfish      -EEAPKPKKAAPAS---KPKKAAKPPK---------PKKAAKSKRT-KTPEKPKKPAPK-
Cone_worm       -AAKKKTAVKPKPK--KTAAADKPKKS---------PKKVKKADGEKKKSSPKKKKADST
Octopus         -AKKEE----KKKT--KTKSAGKTK-----------PKKAKAP----KKVTKSPKKAD--
Jap_fluke       -TVTPKKTVRPKTV--KPKSSPKKKAA---------TKKPKTAKPKSVSSSNPTKPTAAA
Fluke_worm      -SVTPKKVARPKTS--KPKTTP-KKVS---------TKKPKVVKPK--STTNPTSKPAAS
Ch_crab         -DE-PKAVAKKPAKK----AAAKKPVK---------KPAAKKPAAKK----SPKKPAAKK
Coral           -EKKEKKPKKKPAAKKKPAAKPKKPAA---------KKAAKKPAAKK----AAKKPAAKK
Mar_worm        -EKPVTTTKAKKTVKAPAKKAKAKSPA---------KKTAAKPKAKK----TPVKRTAKP
Lucina_H1B      -EKKAA-PKKVVKA-KKPKSPKKKA-----------AKKPAAAGEKKPKAKK-AAKPKKA
Poly_worm       -EVKAEKPKKATKP-KAAAKPKKAA-----------AKKPKAKKSAKTTPKKAAAKPKKA
Cayen_tick      -DKAAAAPKKSPVK-RGPKKAGAAK-----------PKK-VAKPKAAKSPAKKAAKP-KS
Star_tick       -DKAAAGPKKSPVK-RGPKKAGVAK-----------PKKVIMKTTLAKSPAKKAAKP-KA
Gulf_tick       -DKAAAGPKKSPAK-RGPKKAGAAK-----------SPKKAAKPKAAKSP-KKAAKP-KA
Bont_tick       -DKAAAGPKKSPAK-RGPKKAGAAK-----------SPKKAAKPKAAKSP-KKAAKP-KA
Soft_tick       -DKTTASSEKKPKKVAAAKKPAKPK-----------KAASPKKKAAAPKKEKKAATPKKA
Mouro_tick      -DK----VEKAPKKKVAAKKPAAKK-----------SPAKPKAAKKPAAKPKKAASPKKA
Clam            -EAKVVKKKPAKAKKAAKPKAAK-------------PKKAKSTPKKKKPAAKKPAGE-KK
Med_mussel      -EAKVVKKKPAKAKKAAKPKAAK-------------PKKAKSTPKKKKPAAKKPAGE-KK
Mussel          -EAKVVKKKPAKAKKAAKPKAAK-------------PKKAKTTPKKKKPAAKKPAGE-KK
Chile_mus       -EAKQAKKKPAKAKKAAKPKAAK-------------PKKAKSTPKKKK-AAKKPAGE-KK
Cali_mus        -EAKQAKKKPAKAKKAAKPKAAK-------------PKKAKSAPEKKK-AAKEPAGE-KK
Solen_mar       -QAKQAKKKPAKAKSAAKPKAAK-------------PKEAKSAPEKKR-AAKEPARE-KK
Lucina_H1A      -EAK---KKP--AKKAAKPKAAK-------------PKKAKSPKKAKKPAAKKVKKTPKK
Leech           KEEKETKPKKVLAKRAATTKAVKSPAKKVEAAPRKSPKKVKAAETKKKPKASPVKKT-KP
Sponge          -ESKSEK--PSKVKKVAAKKPAAKKA----------STPKK-PKAKKTTTKKTATPKKTK
Owl_snail       -ESKSEK--PSKVKKVAAKKPAAKKA----------STPKK-PKAKKTTTKKTATPKKTK
Sea_hare        -EKSVEKKKPKKDKKPKDKKKSPKKT----------VKPKKAPGTKKTPTKSKKTGDVKK
Oyster          -ESAVKK--PKAKKAAKPKKAAAVK------------KPKKAVTKKPAGEKKAAKPKAKK
Snail           -KEEAKAKKPKAVKKAEKPKAAKPKS----------PAKAKKPKAKKAPTAKKPAAAKKP
Pearl_oyst      ---EAKTKSHKAKPKAEK-KAAKPK-------------KAAAPKGEKK--AKK---AKKP
Scallop         -DKG---KEKPAKKTKKPAAAAATTKK---------AKVDKKKVSKPKKSK------AKT
Centepide       -EKAGPVAKKPAKKAVKAAPPAVEKVK---------VEAVKKPTKTPKKKKKKEIVKVKA
Pig_worm        -EGKKTETVK--AKKPAAKKTTAG-----------KAPVVKKPKSPKKAAVAKPKKAKSS
Roundworm       -EGKKTETVK--AKKPAAKKTTAG-----------KAPVVKKPKSPKKAAVAKPKKAKSS
Dog_worm        -EGKKAETAKKKAKKSAAKKVAGE-----------KKTAAKKPKSPTKAA-AKPKKAKVS
Pine_worm       -DSAPKPKKAPKAKKPAAAKKAPASPKP-------KKAAVKKAAKPKSPAKAKAPKAKKP
Nod_worm        -EKTESTAKP----------------------------KPKAPKTKKEKSAKSPKKASK-
Hookworm        -ESSEIPAKPKVAKIPKAATDEAKPKKA-------TATKPKAPKATKEKSAKSPKKATK-
Pole_worm       -EKAAAPAASKPKK----------------------EKKPKAAGTPKKKAASKPKKAAG-
Cave_fish       PVKKAAPKA---KKPAAKKPAAAKKPK---------KVAAKKPVAAKKSPKKAKKPA-AA
Catfish         PLKKAAPKA---KK-----PAAAKKPK---------KVAAKKP-AAKKSPKKAKKP---A
Zebrafish       AAKKTAAKA---KKPAAKKPAAKKSPKKA------KKPAATSVKKATKSPKKAKKP--AA
Trout           AKKAAAPKA---KKVAAKKPAAAKKPK---------KVAAKKAVAAKKSPKKAKKP--AT
Chicken         KKKP-AAKP---KKPA------AKKPAA-------AAKKPKKAAAVKKSPKKAKKPAAAA
Mallard         KKKP-AAKP---KKPA------AKKPAS-------AAKKPKKAAAVKKSPKKAKKPAAAA
Rabbit          PKKAGAAKP---KKPAG----AAKKPKK-------ATGAATPKKGAKKTPKKAKKPAAAA
Bar_midge       PMKATGEKK--PKA--AAKPKKAGEK---------KKSIAK--KPKAATATKVKKPVAKS
Ten_midge       PKKAPATPK--PKS--TKPKRVTGEKKVV------KKPAAK--KPEAKKATKAAKPAAK-
Fly             PKASAVEKK--TKK--VNASAARATKKKS------STSTTK--KAAGAADKKLSKSAPTK
Arz_bug         KKVPAKTPK--EKK--AAATKAAAPKKK--------TAATS--KKPAAAEKK--KAAAAA
Tarsalis        PKKAAGEKKKVAKK--AAAKKPAGEKKAA------AKKPKAAAAPKKAAVKKVKAATAKT
Albopictus      PKKAAGEKKKAAKK--PAAKKATGEKK--------AKKP---AAKKPAGEKKAKAAGAKA
Gambiae         K-AAAPKKAAAEKKKKTAAKKPAGEKKTV------AKKAT--KKADGAAAKKPKAAAAKK
Mosquito        KKAAAPKKAAAEKKKKTAAKKPAGEKKTV------AKKAT--KKADGAAAKKPKAAAAKK
Tube_worm       PKKPAKKPA--AKK--ATKPKAAAKPK------------------KPKTPKKKAAAKPKK


Mouse           --PVKKAK------KKPAATPKK---------------AKKPKVVKVKPV----KASKPK
Rat             --PVKKAK------KKPAATPKK---------------AKKPKIVKVKPV----KASKPK
Bull            --PVKKAK------KKPAATPKK---------------TKKPKTVKAKPV----KASKPK
Human           --PVKKAK------KKLAATPKK---------------AKKPKTVKAKPV----KASKPK
Orangutan       --PVKKAK------KKLAATPKK---------------AKKPKTVKAKPV----KASKPK
Amz_snake       --AAKKAK------KKPAPAPKK---------------AKKPKTVKAKPV----KASKPK
Cobra           --AAKKAK------KKPAPAPKK---------------AKKPKTVKAKPV----KASKPK
Tas_devil       --AAKKVK------KKAATPAKK---------------AKKPKTVKSKPV----KASKPK
West_frog       KKVKKPAK------KKPAPSPKK---------------AKKTKTVKAKPV----RASRVK
Goose           ---ARKAR------KKSRASPKK---------------AKKPKTVKAKSL----KTSKPK
Salmon          K-KVKKSP------KKAAPKPKK--------------VAKKTK--VAKPA----KATKPK
Sea_louse       K-KVKKSP------KKAAPKPKK--------------VAKKTK--VAKPA----KATKPK
Bow_trout       K-KVKKSP------KKAAPKPKK--------------VAKKTK--VAKPA----KATKPK
Pike_fish       K-KVKKSP------KKVAPKPKK--------------VVKKVK--AAKPA----KAVKPK
Smelt_fish      K-KVKKSPE----KKKAAPKPKK--------------AVKKAK--PTKP-----KASKPK
Gar_fish        K-KAKKAPE----KKKPAPKPKKQE------------KAKKPK--VSKPA----KASRPK
Killifish       --KVKKVA------KKATPAKAKKA------------PAKKSKAAKPKAK----PVKKAA
Turq_killi      --KVKKVA------KKATPAKAKKA------------PAKKSKAAKPKAK----PAKKAA
Blu_notho       --KVKKVA------KKATPAKAKKA------------PAKKSKAAKPKAK----PVKKAA
Seabass         --KVKKVA------KKSTPAKAKKA------------PVKKPKAAKPKAK----PAKKAA
Tilapia         --KVKKVA------KKATPVKAKKA------------PAKKPKAAKPKAK----PAKKAS
Platyfish       --KVKKVA------KKATPAKTKKA------------PAKKPKAAKAKAK----PVKKTA
Molly           --KVKKVA------KKATPAKTKKA------------PAKKPKAAKAKAK----PVKKTA
```

```
Livebearer      --KVKKVA------KKATPAKSKKA------------PVKKPKAAKAKAK----PVKKTA
Stck_fish       --KVKKVA------RKATPAKAKKA------------PAKTP--AKTPAK----KASKAV
Putterfish      --KVKKAP------KKASPAKAKKA------------PPKKSKPVKSKAK----PAKKAA
Cone_worm       TKAVEKAKKPKADKVKKVDKPKKVAKTDKA-------KPKASK--KVKKV----AKTKEP
Octopus         -KKVKKAK---------VAKPVK---------------KPKTPK--KAKTA----AK-KST
Jap_fluke       KPKPTKPK---------AAKPKK---------------AKTPH--KPKTV----KP-KKP
Fluke_worm      KPKATKPKS--------TAKPKK---------------AKSPR--KPKTV----KP-KKP
Ch_crab         --SPKKPA-------AKK-SPKKP--------------AAKKAAKKPAAK----KAAKKP
Coral           P-AAKKPA-------AKKPAAKKP--------------AAKKPAKKPAAK----KPAKKP
Mar_worm        --KTKKSP-------KKAAKPKKA--------------AAKKTAQKKAAA----KP-KKA
Lucina_H1B      K--SPKK--------AAKPKKAK--------------TPKKAGAKA-------S-KPKTK
Poly_worm       AKKSPKK--------AATKKAAG--------------KPKAAAAKK-------T-PKKAA
Cayen_tick      PKAAKPKT------AAAKPKVAK--------------KPKSPKKAK-------S-PKKPK
Star_tick       PKAAKPKT------AAAKPKVAK--------------KPKSPKKAK-------S-PKKPK
Gulf_tick       AKAAKPK-------AAAKPKAAK--------------KPKAPKKAK-------S-PKKPK
Bont_tick       AKAAKPKT------AAAKPKVAK--------------KPKAPKKAK-------S-PKKPK
Soft_tick       P-KAKATK------AAAKPKAAP--------------KPKTPKKAK-------S-PKKPK
Mouro_tick      AAKPKAAT------KAAKPKAAA--------------KPKTPRKTKPVKKA--S-PKKPK
Clam            AAKPKAK-------KPAAKKAAK--------------PKKPAAKSPAKKK-----AAKPK
Med_mussel      AAKPKAK-------KPAAKKAAK--------------PKKPAAKSPAKKK-----AAKPK
Mussel          AAKPKAK-------KPAAKKAAK--------------PKKPAAKSPAKKK-----AAKPK
Chile_mus       AAKPKAK-------KPAAKKAAK--------------PKK-AARSPAKKK-----AAKPK
Cali_mus        AAKPKAK-------KPAAKKGAK--------------AKKAAPRSPAKKK-----AAKPK
Solen_mar       AAKPKAL-------KPAAKKVAK--------------AKKAAPRSPAKKK-----AAKPK
Lucina_H1A      AVKPKK-----------ATKSPK--------------KAKKATKSPKKAK-----ASKPK
Leech           AAKPKAAS------KPPAAKPAKKE------------VKKSPAKPKAKAA-----ASPAK
Sponge          VAGAKKTA------AKPKKAAPK--------------KVKTPKKVKTVK-------PKKA
Owl_snail       VAGAKKTA------AKPKKAAPK--------------KVKTPKKVKTVK-------PKKA
Sea_hare        VT-KKATG------AKTKKATTKSPG-----------KKNSPAKKSTAAK----KVAKKA
Oyster          AKSPKKAK------AAGEKKPKK--------------VAKSPAKAKKAP-------AKPK
Snail           KSPKKAA--------VKKPKAPK---------------PAAAAKPKKPKT----PKKKAA
Pearl_oyst      KPAKSKP--------AKKVKTPK---------------KAAAKKPK----------KAAK
Scallop         EKKAKASP---------KKKAAAKPK-----------KAKSPKKAAAK--------SKAK
Centepide       VKKAKSPK------KVVEKKKTVKAK-----------KVKTPKKAVIKKP-----VSKPK
Pig_worm        PKKAAAK-------PKKAVSPKK--------------PKASKPK-----------TVKKS
Roundworm       PKKAAAK-------PKKAVSPKK--------------PKASKPK-----------TVRKS
Dog_worm        PKKAAAK-------PKKAASPKK--------------PKVAKPK-----------APKKS
Pine_worm       AAAAKPKS------PAKKAAPKK--------------AVVKKS------------PAKKA
Nod_worm        PKAKSAKS------PKKAATPKK--------------AASKPK------------TAKKP
Hookworm        PKAKTAKS------PKKAATPKK--------------AAPKPK------------ATKKT
Pole_worm       DKPKKAKI------AKKSKSPKK--------------AAAKPKKAKSPKKPAVKKAAKKP
Cave_fish       AKKATKSPK----KVKKPAAPKK--------------ATKSPKKAKTVKP-------KAA
Catfish         AKKATKSPK----KAKKPATPKK--------------AAKSPKKAKAVKP-------KTT
Zebrafish       AKKATKSPK----KAKKPAAAKK--------------AAKSPKKVKAVKP-------KTA
Trout           PKKAAKSPK----KATKAAKP-K--------------AAKPKKAAKSPK-----------
Chicken         TKKAAKSPK----KATKAGRPKK--------------TAKSPAKAKAVKP-------KAA
Mallard         TKKAAKSPK----KAAKAGRPKK--------------AAKSPAKAKAVKP-------KAA
Rabbit          GAKKAKSPK----K-AKAAKPKK--------------APKSPAKAKAVKP-------KAA
Bar_midge       TKKQAAVKP----AAKK-AAPKP-------------KAVPKPK-AAKPK--------KEA
Ten_midge       ---KVAAKP----AAKKAAAPKP-------------KAAAKPKKEVKPK--------KEA
Fly             KSVEKKRAD----KAKAKDAKKTGTIKAKPTTAKAKSSATKPK-TPKPKT-------KSA
Arz_bug         VAVKTKTPS----KAK-----KT-----------AQPPTKKPK-SPKPKK-------AAV
Tarsalis        AKKAG--------TVKKAAAPKQ-------------KATKPSKTAAK-KP-------KTP
Albopictus      AKKAG--------TVKKAAAPKQ-------------KATKPSKAAAK-KP-------KTP
Gambiae         PKAADGAKK----AAKKPAAPKQ-------------KATKPAKAAAAAKP-------KAP
Mosquito        PKAADGAKK----AAKKPAAPKQ-------------KATKPAKAAAAAKP-------KAP
Tube_worm       EKKEKKAKT----PKKAKAAVKK--------------TPKKAAAKPKTVK-------KAA


Mouse           KAKTVKPK-AK-SSAKRASKKK-------------
Rat             KAKPVKPK-AK-SSAKRASKKK-------------
Bull            KTKPVKPK-AK-SSAKRTGKKK-------------
Human           KAKPVKPK-AK-SSAKRAGKKK-------------
Orangutan       KAKPVKPK-AK-SSAKRAGKKK-------------
Amz_snake       KAKASKPK-AK-SSAKKSTKKK-------------
Cobra           KAKASKPK-AK-SSAKKSTKKK-------------
Tas_devil       KAKPSKPK-AK-SSAKKSAKKK-------------
West_frog       KAKPSKPK-AK-ASPKKSGRKK-------------
Goose           KARRSKPR-AK-SGARKSPKKK-------------
Salmon          KAKAAKPK-PK-AAAKKAAKKK-------------
Sea_louse       KAKAAKPK-PK-AAAKKAAKKK-------------
Bow_trout       KAKAAKPK-PK-AAAKKAAKKK-------------
Pike_fish       KAKAAKPK-PK-AAAKKAAKKK-------------
Smelt_fish      KAKTAKPK-PK-TTAKKATKKK-------------
Gar_fish        KMKTAK-----------------------------
Killifish       KPKAKTPK-KA-AKS---AKKK-------------
Turq_killi      KPKAKTPK-KA-AKS---AKKK-------------
Blu_notho       KPKAKTPK-KA-AKS---AKKK-------------
Seabass         KPKAKPAK-KA-AKT---AKKK-------------
Tilapia         KPKAKTPK-KA-AKT---SKKK-------------
Platyfish       KPKAKTPK-KA-AKT---SKKK-------------
Molly           KPKAKTPK-KA-AKT---SK---------------
Livebearer      KSKAKTPK-KA-AKT---SKKK-------------
Stck_fish       KSKAKPAR-RV-AKPKGKAPKKG------------
Putterfish      KPKAKPAK-KA-TKT---AKKK-------------
Cone_worm       KTKAPKAK-KVLKTPKKSASKK-------------
Octopus         KSKP---------KPKKSASKK-------------
Jap_fluke       VAKK---------TPKKVAKK--------------
Fluke_worm      VAKK---------TPKKVAKK--------------
```

```
Ch_crab          AAKK---------AAKK------------------
Coral            AKKP---------AAKKTAKK--------------
Mar_worm         VKKP---------AAKKTAAKKK------------
Lucina_H1B       -KKSPKK--------AKAARK--------------
Poly_worm        PKKKPAA--------KKAAKKAPAKK---------
Cayen_tick       VAKKATP--------KKAAAKK-------------
Star_tick        VAKKATP--------KKAAAKK-------------
Gulf_tick        VAKKASP--------KKAAAKK-------------
Bont_tick        VAKKATP--------KKAAKK--------------
Soft_tick        -AKKAPA--------KKAAAKK-------------
Mouro_tick       -AKKATP--------AKKSARK-------------
Clam             AKKTPK---------KK------------------
Med_mussel       AKKTPK---------KK------------------
Mussel           AKKTPK---------KK------------------
Chile_mus        AKKTPK---------KK------------------
Cali_mus         AKKTPK---------KK------------------
Solen_mar        AKKTPK---------NK------------------
Lucina_H1A       KSKTPK---------KKATKK--------------
Leech            AKKAAKPA-AK---PKKAAKK--------------
Sponge           SPK------------KKTAAKKA------------
Owl_snail        SPK------------KKTAAKKA------------
Sea_hare         APKTTKP--------KKAAATKK------------
Oyster           AKKAAKP--------KKAAAQK-------------
Snail            APKKAAG-------AKKAAAKKEK-----------
Pearl_oyst       SPKKAKK-------AAKA-----------------
Scallop          KGKAAK----------K------------------
Centepide        KLKSPK----------KSRK---------------
Pig_worm         APK---------------AKKAAA-----------
Roundworm        APK---------------AKKAAA-----------
Dog_worm         APR---------------AKKAAA-----------
Pine_worm        APKKK------------AAPKAKA-----------
Nod_worm         AAKKA------------AAPAKA------------
Hookworm         APKK-------------AAPAKS------------
Pole_worm        TAKKA------------AAPKA-------------
Cave_fish        K--------PKAAKPKKAAPKKK------------
Catfish          K--------IKAAKAKKAAPKKK------------
Zebrafish        K--------PKAAKPKKAAPKKK------------
Trout            -------------KVKKPAAAKK------------
Chicken          KSKAAK---PKAAKAKKAATKKK------------
Mallard          KPKAAK---PKAAKAKKAAPKKK------------
Rabbit           KPKAAK---PKTAKPKKAPAKKK------------
Bar_midge        KPKKAA----APKAAKKPAQKKPKATKKPAAKKA-
Ten_midge        KPKKAA----A-KPAKKPAAKP---AKKPAAKKAK
Fly              KPKKVV----SATTPKKTAVKKPKAKTASATKK--
Arz_bug          K---------KAKTPKKAAPNKK------------
Tarsalis         KPK-------KAAAPKKAAAKK-------------
Albopictus       KPK-------KAAPAKKAAPKKAAAKSKK------
Gambiae          KPKKAAAPAKKAAAPKKAAAPKKAAAPKKAAAAKK
Mosquito         KPKKAAAPAKKAAAPKKAAAPKKAAAPKKVAAAKK
Tube_worm        KPKT------PKKAAKKPAAKKSK-----------
```

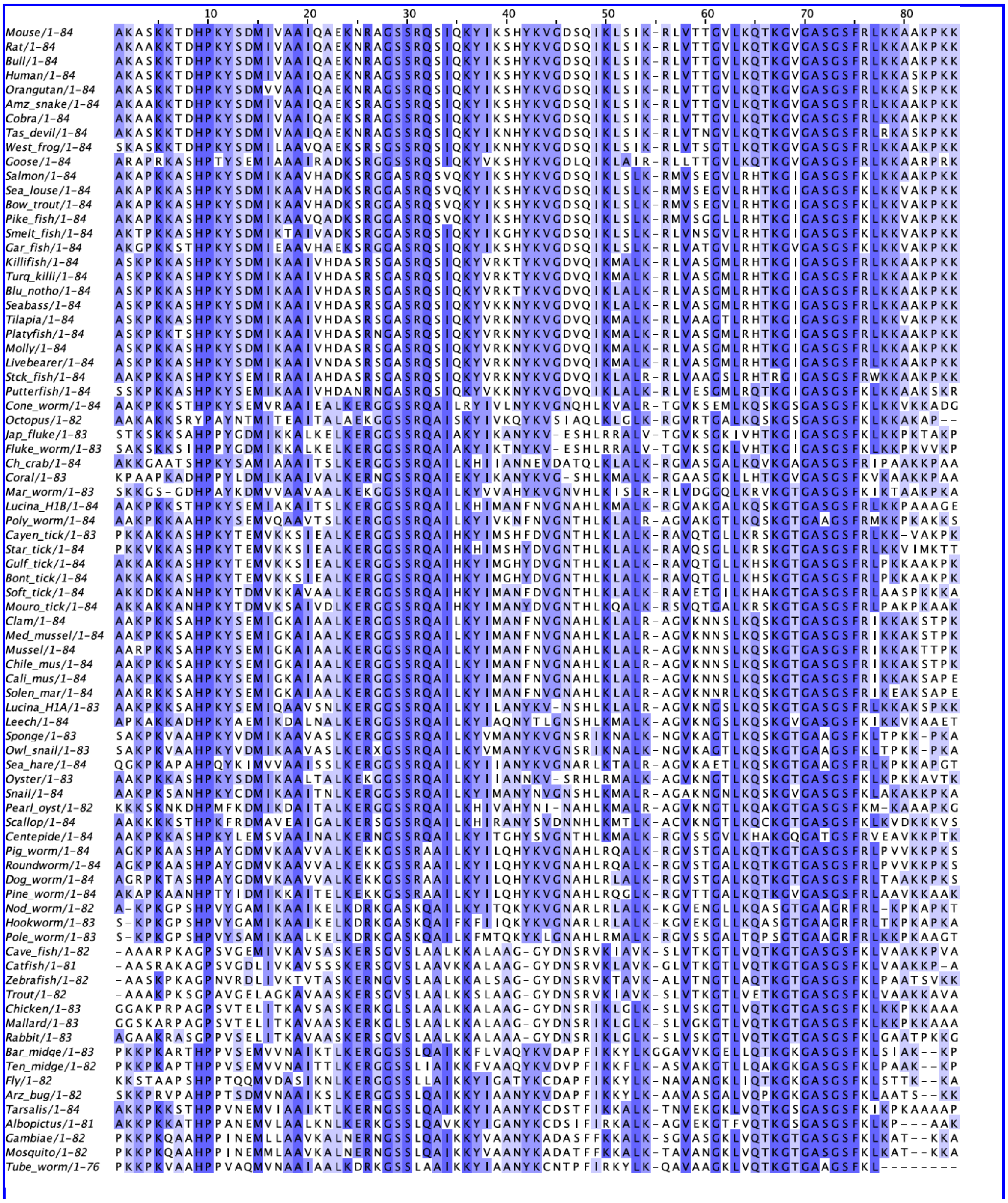

Figure S2. Multiple sequence alignment of the conserved central globular domain of histone H1s from 80 different species. Highly conserved amino acid residues are shown in dark blue and the level of shading decreases for those residues that are less conserved. Mammalian sequences are at the top, followed by reptiles, marsupial, amphibian, bird, fish, worms, cephalopod, crab, clam, insects, mussels, etc.

Table S1. Complete List Of H1 Protein Sequences Used For The Multiple Sequence and Phylogenetic Analyses.

Entry	Organism ID	Phylogenetic Abbreviation
Q6NVM0	Xenopus tropicalis (Western clawed frog) (Silurana tropicalis)	West_frog

P02258	Anser anser anser (Western greylag goose)	Goose
P02254	Salmo trutta (Brown trout)	Trout
P10922	Mus musculus (Mouse)	Mouse
P09987	Gallus gallus (Chicken)	Chicken
P43278	Rattus norvegicus (Rat)	Rat
Q0IIJ2	Bos taurus (Bovine)	Bull
P07305	Homo sapiens (Human)	Human
Q5NVN9	Pongo abelii (Sumatran orangutan) (Pongo pygmaeus abelii)	Orangutan
P02252	Oryctolagus cuniculus (Rabbit)	Rabbit
P09426	Anas platyrhynchos (Mallard) (Anas boschas)	Mallard
P40264	Glyptotendipes barbipes (Midge) (Chironomus barbipes)	Bar_midge
P40278	Chironomus tentans (Midge) (Camptochironomus tentans)	Ten_midge
Q94555	Drosophila virilis (Fruit fly)	Fly
B3FEA3	Ruditapes Philippinarum (Japanese littleneck clam) (venerupis philippinarum)	Clam
O46140	Mytulis edulis (Blue mussel)	Mussel
Q7K957	Mytulis galloprovincialis (Mediterranean mussel)	Med_mussel
Q8MYC2	Mytulis chilensis (Chilean blue mussel)	Chile_mus
Q8MYC3	Mytulis californianus (California mussel)	Cali_mus
K1RJJ7	Crassostrea gigas (Pacific oyster) (Crassostrea angulata)	Oyster
D2DLE5	Solen marginatus	Solen_mar
A0A2C9K5S1	Biomphalaria glabrata (Bloodfluke planorb) (Freshwater snail)	Snail
X1ZAF3	Capitella teleta (Polychaete worm)	Poly_worm
A0A023FRV8	Amblyomma cajennense (Cayenne tick)	Cayen_tick
A0A1X7T2H6	Amphimedon queenslandica (Sponge)	Sponge
G3MGS5	Amblyomma maculatum (Gulf Coast tick)	Gulf_tick
V4AZD7	Lottia gigantea (Giant owl limpet)	Owl_snail
F0JA34	Amblyomma variegatum (Tropical bont tick)	Bont_tick
A0A194ANP8	Pinctada fucata (akoya pearl oyster) (Pinctadaimbricata fucata)	Pearl_oyst
I1SKI8	Aplysia californica (California sea hare)	Sea_hare
A0A1Z5KW53	Ornithodoros moubata (Soft tick) (Argasid tick)	Soft_tick
A0A1D2AHZ2	Ornithodoros brasiliensis (Mouro tick)	Mouro_tick
A0A0K1R009	Pectinaria gouldii (Trumpet worm) (ice-cream cone worm)	Cone_worm
A0A210PEH9	Mizuhpecten yessoensis (Japanese scallop) (Patinopecten yessoensis)	Scallop
T1G7G6	Helobdella robusta (Californian leech)	Leech
B9ELA2	Salmo salar (Atlantic salmon)	Salmon
C1BK29	Osmerus mordax (Rainbow smelt) (Atherina mordax)	Smelt_fish
A0A060VV51	Oncorhyncus mykiss (Rainbow trout) (Salmo gairdneri)	Bow_trout
C1BNT2	Caligus rogercresseyi (Sea louse)	Sea_louse
F1LAA1	Ascaris suum (Pig roundworm) (Ascaris lumbricoides)	Pig_worm
A0A0B2V393	Toxocara canis (canine roundworm)	Dog_worm

A0A0K0Q063	Eriocheir sinensis (Chinese mitten crab)	Ch_crab
A0A0M3IK88	Ascaris lumbricoides (Giant roundworm)	Roundworm
A0A0C9RTG1	Amblyomma americanum (Lone star tick)	Star_tick
O18642	Chaetopterus variopedatus (Parchment tube worm) (Tricoelia variopedata)	Tube_worm
C1BYR7	Esox lucius (Northern pike)	Pike_fish
F4ZHB6	Astyanax fasciatus (Blind cave fish) (Astyanax mexicanus)	Cave_fish
A0A140LH24	Danio rerio (Zebrafish) (Branchydanio rerio)	Zebrafish
Q4S4R9	Tetraodon nigroviridis (Spotted green pufferfish) (Chelonodon nigroviridis)	Pufferfish
C3Z7D2	Branchiostoma floridae (Florida lancelet) (Amphioxus)	Lancelet
A0A0B1RT83	Oesophagostonum dentatum (Nodular worm)	Nod_worm
A0A2B4S3T9	Stylophora pistillata (Smooth cauliflower coral)	Coral
A0A0L8GWZ4	Octopus bimaculoides (California two-spotted octopus)	Octopus
U6P4D5	Haemonchus contortus (Barber pole worm)	Pole_worm
A0A2D4N2X1	Nicrurus spixii (Amazon coral snake)	Amz_snake
V8PA09	Ophiophagus hannah (King cobra) (Naja hannah)	Cobra
A0A1A8QW5	Nothobranchius rachovii (bluefin notho)	Blu_notho
G3P2U9	Gasterosteus aculeatus (Three-spined stickleback)	Stck_fish
D2N2Q1	Xenoturbella bocki (Marine worm)	Mar_worm
A0A1A8KG98	Nothobranchius kuhntae (Bera killifish)	Killifish
A0A1A8V9N7	Nothobranchius furzeri (Turquoise killifish)	Turq_killi
W2TF07	Necator americanus (Human hookworm)	Hookworm
A0A1Q3EVA2	Culex tarsalis (Encephalitis mosquito)	Tarsalis
C1LBZ2	Schistosoma japonucum (Blood fluke)	Jap_fluke
G4V6K8	Schistosoma mansoni (Blood fluke)	Fluke_worm
A0A1B6CN64	Clastoptera arizonana (arizona spittle bug)	Arz_bug
A0A1S4H614	Anpheles gambiae (African malaria mosquito)	Gambiae
A0A023EJZ9	Aedes albopictus (Asian tiger mosquito) (Stegomyia albopicta)	Albopictus
A0A182HFW6	Anopheles arabiensis (Mosquito)	Mosquito
A0A17RTC3	Bursaphelenchus xylophilus (Pinewood nematode worm) (Aphelenchoides xylophilus)	Pine_worm
G3W6X3	Sarcophilus harrisii (Tasmanian devil) (sarcophilus laniarius)	Tas_devil
M4B0V9	Xiphophorus maculatus (Southern platyfish) (Platypoecilus maculatus)	Platyfish
A0A2DORM10	Ictalurus punctatus (Channel catfish) (silurus punctatus)	Catfish
I3KZG4	Oreochromis niloticus (Nile tilapia) (Tilapia nilotica)	Tilapia
A0A087YRU6	Poecilia formosa (Amazon molly) (Limia fromosa)	Molly
W5NNJ0	Lepisosteus oculatus (Spotted gar)	Gar_fish
E6ZGX2	Dicentrarchus labrax (European seabass) (Morone labrax)	Seabass
T1JEZ9	Strigamia maritima (European centepide) (Geophilus maritimus)	Centepide

A0A0S7KUC6	Poeciliopsis prolifica (blackstripe livebearer)	Livebearer
